# Genome Data Provides High Support for Generic Boundaries in *Burkholderia* Sensu Lato

**DOI:** 10.3389/fmicb.2017.01154

**Published:** 2017-06-26

**Authors:** Chrizelle W. Beukes, Marike Palmer, Puseletso Manyaka, Wai Y. Chan, Juanita R. Avontuur, Elritha van Zyl, Marcel Huntemann, Alicia Clum, Manoj Pillay, Krishnaveni Palaniappan, Neha Varghese, Natalia Mikhailova, Dimitrios Stamatis, T. B. K. Reddy, Chris Daum, Nicole Shapiro, Victor Markowitz, Natalia Ivanova, Nikos Kyrpides, Tanja Woyke, Jochen Blom, William B. Whitman, Stephanus N. Venter, Emma T. Steenkamp

**Affiliations:** ^1^Department of Microbiology and Plant Pathology, Forestry and Agricultural Biotechnology Institute, University of Pretoria Pretoria, South Africa; ^2^DOE Joint Genome Institute, Walnut Creek CA, United States; ^3^Bioinformatics and Systems Biology, Justus-Liebig-University Giessen Giessen, Germany; ^4^Department of Microbiology, University of Georgia, Athens GA, United States

**Keywords:** *Burkholderia*, *Paraburkholderia*, *Caballeronia*, phylogenomics, *Robbsia andropogonis*, *Burkholderia rhizoxinica*

## Abstract

Although the taxonomy of *Burkholderia* has been extensively scrutinized, significant uncertainty remains regarding the generic boundaries and composition of this large and heterogeneous taxon. Here we used the amino acid and nucleotide sequences of 106 conserved proteins from 92 species to infer robust maximum likelihood phylogenies with which to investigate the generic structure of *Burkholderia* sensu lato. These data unambiguously supported five distinct lineages, of which four correspond to *Burkholderia* sensu stricto and the newly introduced genera *Paraburkholderia*, *Caballeronia*, and *Robbsia*. The fifth lineage was represented by *P. rhizoxinica*. Based on these findings, we propose 13 new combinations for those species previously described as members of *Burkholderia* but that form part of *Caballeronia*. These findings also suggest revision of the taxonomic status of *P. rhizoxinica* as it is does not form part of any of the genera currently recognized in *Burkholderia* sensu lato. From a phylogenetic point of view, *Burkholderia* sensu stricto has a sister relationship with the *Caballeronia*+*Paraburkholderia* clade. Also, the lineages represented by *P. rhizoxinica* and *R. andropogonis*, respectively, emerged prior to the radiation of the *Burkholderia* sensu stricto+*Caballeronia*+*Paraburkholderia* clade. Our findings therefore constitute a solid framework, not only for supporting current and future taxonomic decisions, but also for studying the evolution of this assemblage of medically, industrially and agriculturally important species.

## Introduction

The genus *Burkholderia* was originally introduced to accommodate an assemblage of seven *Pseudomonas* species (Yabuuchi et al., 1992), two of which were later transferred to *Ralstonia* (Gillis et al., 1995; Yabuuchi et al., 1995). Since then, the number of *Burkholderia* species has grown substantially, to about 108 in 2015 (Estrada-de los Santos et al., 2016), spanning a range of human, animal and plant pathogens, as well as numerous strains with significant biotechnological potential (Depoorter et al., 2016; Estrada-de los Santos et al., 2016). The latter includes the so-called plant beneficial and environmental (PBE) species (Suárez-Moreno et al., 2012), many of which are plant-associated (e.g., those with plant growth promoting activities, the symbiotic diazotrophs and free-living species with diazotrophic, bioremedial and antibiotic activities) (Depoorter et al., 2016; Estrada-de los Santos et al., 2016). Because of this heterogeneity, new genera [e.g., ‘*Caballeronia’* (Gyaneshwar et al., 2011) and ‘*Paraburkholderia’* (Sawana et al., 2014)] has been introduced to accommodate most of the PBE species (Oren and Garrity, 2015a,b, 2017), while retaining the pathogens in *Burkholderia* sensu stricto. Most recently, a third genus, *Robbsia* was introduced to accommodate the phytopathogen previously referred to as *B. andropogonis* (Lopes-Santos et al., 2017).

Overall, the taxonomy of *Burkholderia* sensu lato remains in significant flux (Estrada-de los Santos et al., 2016). With their review of the group, Estrada-de los Santos et al. (2016) recognized two monophyletic groups [Groups A and B; A consists of *Caballeronia* and *Paraburkholderia* as circumscribed by Gyaneshwar et al. (2011) and Sawana et al. (2014), respectively, while Group B includes most of the notable human, animal and plant pathogens, as well as the so-called “*B. cepacia* complex”]. They showed that *B. andropogonis* (now *Robbsia andropogonis*) is separated into its own group, and they designated two so-called “Transition Groups” (i.e., 1 and 2; neither were supported as monophyletic and both contained mainly environmental species). Since then, Dobritsa and Samadpour (2016) have proposed the transfer of species in Transition Group 2 to a new genus. However, to complicate the issue, this new genus was named “*Caballeronia”* although its proposed usage is not synonymous with the one previously proposed by Gyaneshwar et al. (2011) for accommodating all the PBE isolates.

The proposals to split *Burkholderia* sensu lato were based almost entirely on evidence from 16S ribosomal RNA (rRNA) phylogenetic trees with limited and in some cases no statistical support (Gyaneshwar et al., 2011; Dobritsa and Samadpour, 2016; Eberl and Vandamme, 2016). Even phylogenies based on conventional multilocus sequence analysis (MLSA) using the combined sequence information for 4–7 genes (Gevers et al., 2005) produced phylogenies in which the major groups were not supported as monophyletic (Estrada-de los Santos et al., 2013). Also, the most comprehensive phylogenetic hypothesis to date (based on 21 conserved gene sequences) lacked sufficient representation across this diverse assemblage (Sawana et al., 2014). Thus, uncertainties remain regarding the genomic and evolutionary coherence of *Burkholderia* sensu lato and its lineages. This, in turn, blurs the boundaries of the *Burkholderia* sensu lato genera currently recognized and also casts doubt on the appropriateness and legitimacy of their taxonomic circumscriptions.

In this study, we aimed to resolve the relationships within *Burkholderia* sensu lato, particularly those pertaining to *Paraburkholderia* and *Caballeronia*, by making use of whole genome sequence data. For this purpose, we utilized all of the sequences for type strains (or appropriate representatives) available in the public domain. To increase representation of the so-called environmental species, we also determined the sequences for eight additional taxa via Phase III of the GEBA (Genomic Encyclopedia of Bacterial and Archaeal type strains) project (Whitman et al., 2015). These included the rhizobial species *P. aspalathi* (Mavengere et al., 2014) and *P. diazotrophica* (Sheu et al., 2013), and the soil bacteria *P. hospita* (Goris et al., 2002), *P. phenazinium* (Viallard et al., 1998), *P. sartisoli* (Vanlaere et al., 2008), *P. terricola* (Goris et al., 2002), as well as the plant-associated diazotrophic species *P. caballeronis* (Martínez-Aguilar et al., 2013) and *P. tropica* (Reis et al., 2004).

## Materials and Methods

### Whole-Genome Sequencing of Eight *Paraburkholderia* Type Strains

The eight type strains (*P. aspalathi* LMG 27731^T^, *P. hospita* LMG 20598^T^, *P. diazotrophica* LMG 206031^T^, *P. phenazinium* LMG 2247^T^, *P. sartisoli* LMG 24000^T^, *P. terricola* LMG 20594^T^, *P. tropica* LMG 22274^T^ and *P. caballeronis* LMG 26416^T^) were obtained from the Belgian Coordinated Collections of Microorganisms (University of Gent, Belgium). Routine growth of these bacteria in the laboratory and extraction of high quality genomic DNA were completed as described previously (Steenkamp et al., 2015). Whole genome sequencing was performed by the Joint Genome Institute (JGI) following standard protocols^1^ and using the Illumina HiSeq-2500 1TB platform with an Illumina 300 base pair (bp) insert standard shotgun library.

All raw sequences were filtered using BBDuk (Bushnell, 2017), which removes known Illumina artifacts, and PhiX. Reads with more than one “N” or with quality scores (before trimming) averaging less than 8 or reads shorter than 51 bp (after trimming) were discarded. The remaining reads were mapped to masked versions of human, cat and dog reference sequences using BBMap (Bushnell, 2017) and discarded if identity values exceeded 93%. The remaining reads were then assembled into contigs using Velvet version 1.2.07 (Zerbino and Birney, 2008) (the settings used were velveth: 63 –shortPaired and velvetg: –very clean yes –exportFiltered yes –min contig lgth 500 –scaffolding no –cov cutoff 10). The Velvet contigs were then used to generate 1–3 kbp simulated paired end reads using wgsim version 0.3.0^2^ (the settings used were –e 0 –1 100 –2 100 –r 0 R 0 –X 0). We then assembled the quality filtered Illumina reads with the simulated read pairs using Allpaths-LG version r46652 (Gnerre and MacCallum, 2011) (the settings used were PrepareAllpathsInputs: PHRED 64 = 0 PLOIDY = 1 FRAG COVERAGE = 125 JUMP COVERAGE = 25 LONG JUMP COV = 50 and RunAllpathsLG: THREADS = 8 RUN = std shredpairs TARGETS = standard VAPI WARN ONLY = True OVERWRITE = True).

The standard JGI microbial genome annotation pipeline (Huntemann et al., 2015) was used to predict and annotate genes in each of the eight assembled genomes. For this purpose, we specifically used the Prodigal algorithm to identify protein-coding genes (Hyatt et al., 2010). Additional annotation was performed using JGI’s Integrated Microbial Genomes (IMG) system (Markowitz et al., 2014).

### Sequence Datasets and Multiple Alignments

Protein-coding gene datasets were generated for the eight bacteria sequenced here, as well as all the *Burkholderia* sensu lato type strains (or suitable conspecific strains) for which whole genome sequences were available (Supplementary Table S1). This was achieved by using the EDGAR (Efficient Database framework for comparative Genome Analyses using BLAST score Ratios) server^3^ (Blom et al., 2016) to identify single-copy orthologous genes shared among all of the genomes examined. The respective amino acid and nucleotide sequences for each gene dataset were then batch-aligned using the Multiple Sequence Comparison by Log-Expectation (MUSCLE) (Edgar, 2004) iteration-based alignment tool implemented in CLC Main Workbench 7.6 (CLC Bio).

Individual alignments were manually curated in BioEdit version 7.2.5 (Hall, 2011), during which we discarded those genes for which one or more taxa contained more than 5% missing data. The pair-wise protein similarity for the remaining genes (i.e., those for which the datasets were ≥95% complete) were individually determined with Geneious v. 6.1 (Biomatters Limited^4^), followed by concatenation with FASconCAT-G v. 1.02 (Kuck and Longo, 2014). The total pair-wise similarity among the various taxa included in the study was also calculated by making use of the concatenated nucleotide and amino acid datasets using Geneious v. 6.1.

We also evaluated the genomic distribution and functional roles for the genes with ≥95% complete sequence data. The putative function of each gene product was inferred using the Kyoto Encyclopaedia of Genes and Genomes (KEGG) databases and the GhostKoala mapping tool^5^ (Kanehisa et al., 2016), as well as through comparison with the annotated genome of the type species *Burkholderia cepacia* ATCC 25416^T^ (Yabuuchi et al., 1992). This genome was also used to determine the relative genomic position of each gene used in our dataset. This was done by making use of Geneious v. 6.1 and the publicly available annotations of the ATCC 25416^T^ genome on the National Center for Biotechnology Information (NCBI^6^) website.

The level of substitution saturation in the various nucleotide and amino acid datasets were evaluated as described before (Palmer et al., 2017). For this purpose, distances based on actual substitutions (p-distance) were compared to those inferred using an appropriate substitution model (Jeffroy et al., 2006; Philippe et al., 2011). The modeled distances for the nucleotide data were inferred using the General Time Reversible (GTR) substitution model (Tavaré, 1986) and the minimum-evolution distance algorithm (Desper and Gascuel, 2002). Both the p- and GTR-distances were determined in DAMBE v. 6.0.1 (Xia and Xie, 2001) and were calculated for the full nucleotide datasets and for the third codon positions only. For the amino acid datasets, MEGA v.6.06 (Tamura et al., 2013) was used to calculate the p-distances and those based on the Jones-Taylor-Thornton (JTT) model (Jones et al., 1992). Graphical representations of the correlation between the respective distances for each dataset were constructed in Microsoft Excel 2013, followed by linear regression analyses.

### Phylogenetic Analyses

The respective nucleotide and amino acid alignments for the ≥95% complete protein-coding genes were concatenated and subjected to maximum likelihood phylogenetic analyses with RAxML v. 8.2.1 (Stamatakis, 2014). For this purpose, the sequences were concatenated and partitioned using FASconCAT-G. For the amino acid data, each partition employed the best-fit substitution model as indicated by ProtTest v. 3.4 (Abascal et al., 2005). For the nucleotide data, we used the GTR model with independent parameter estimation for each partition. Branch support was estimated in RAxML using the estimated model parameters, the rapid hill-climbing algorithm and non-parametric bootstrap analyses of 1000 repetitions.

## Results

### Whole-Genome Sequences for Eight Type Strains of *Paraburkholderia*

Illumina sequencing allowed assembly of high-coverage (i.e., 67.4 to 119.3 X) draft genomes for the type strains of eight *Paraburkholderia* species (**Table 1**). The number of contigs for each genome ranged from 22 to 188 where more than 50% of the individual genomes were incorporated into relatively large contigs (i.e., respective N50-values ranged from 144482 to 573607). The assembled genomes ranged in size from 5.9 for *P. sartisoli* LMG 24000^T^ to 11.2 million bases for *P. hospita* LMG 20498^T^. The number of genes predicted for each genome also corresponded well with their overall sizes (e.g., 5407 genes were predicted for *P. sartisoli* LMG 24000^T^ and 10534 for *P. hospita* LMG 20498^T^). The G+C content for the eight species ranged from 61.09% for *P. aspalathi* to 67.03% for *P. caballeronis*. The assembled genome sequences for all eight species are available from NCBI (see **Table 1** for accession numbers). Overall the sizes and GC content were comparable to previously sequenced genomes of other Burkholderia sensu lato species (**Table 2**).

**Table 1:** Details regarding the eight *Paraburkholderia* type strains sequenced at JGI for the GEBA Phase III project.

Type strain^a^ (Strain number)	N50 (bp)	Number of contigs	Largest contig size (bp)	Average contig size (bp)	Genome size (bp)	Sequencing depth	G+C (%)	Number of genes	Gold ID	NCBI BioProject Accession
*P. hospita* (LMG 20598)	141903	188	624080	59577	11200469	73.3X	61.87	10534	Gp0116482	PRJNA323250
*P. diazotrophica* (LMG 26031)	180974	112	586763	77577	8688577	102.3X	62.58	8041	Gp0116479	PRJNA323247
*P. phenazinium* (LMG 2247)	342088	52	868734	165344	8597887	67.4X	62.34	7927	Gp0116485	PRJNA323253
*P. sartisoli* (LMG 24000)	573607	22	1593786	269570	5930529	197.0X	63.52	5407	Gp0116487	PRJNA323254
*P. terricola* (LMG 20594)	144482	114	380545	64223	7321401	94.4X	63.62	6748	Gp0116489	PRJNA323256
*P. tropica* (LMG 22274)	341815	61	531529	140659	8580172	119.3X	64.77	7611	Gp0116490	PRJNA323257
*P. caballeronis* (LMG 26416)	339592	42	770885	168261	7066941	104.1X	67.03	6258	Gp0116481	PRJNA323249
*P. aspalathi* (LMG 27731)	216058	103	588940	96042	9892286	112.6X	61.09	9038	Gp0116483	PRJNA323251

^a^*LMG = Belgian Coordinated Collections of Microorganisms, Laboratorium voor Microbiologie, Universiteit Gent*.

**Table 2:** Genome properties for all the investigated species forming part of *Burkholderia* sensu stricto, *Caballeronia* and *Paraburkholderia*.

Species^a^	Size (Mb)	G+C (mol%)	Source
*P. caledonica*	7.3	62	Soil
*P. bryophila*	7.4	62	Moss
*P. kirstenboschensis*	8.3	62	Root nodules
*P. dilworthii*	7.7	62	Root nodules
*P. phenoliruptrix*	7.8	63	Chemostat
*P. graminis*	7.5	63	Plant roots
*P. terricola*	7.3	64	Soil
*P. ginsengisoli*	6.5	64	Soil
*P. monticola*	7.9	64	Soil
*P. tuberum*	9.0	63	Root nodules
*P. sprentiae*	7.8	63	Root nodules
*P. ginsengiterrae*	8.5	63	Soil
*P. xenovorans*	9.7	63	Soil
*P. phytofirmans*	8.2	62	Plant roots
*P. aspalathi*	9.9	61	Root nodules
*P. fungorum*	8.7	62	Fungus
*P. phenazinium*	8.6	62	Soil
*P. sartisoli*	5.9	64	Soil
*P. diazotrophica*	8.7	63	Root nodules
*P. phymatum*	8.7	62	Root nodules
*P. caribensis*	9.0	63	Soil
*P. terrae*	9.9	62	Soil
*P. hospita*	11.2	62	Soil
*P. kururiensis*	6.8	64	Water
*P. caballeronis*	7.1	67	Soil
‘*P. acidipaludis*’	6.5	65	Plant stem
*P. ferrariae*	7.9	65	Iron ore
*P. heleia*	8.0	65	Plant tissue
*P. nodosa*	9.5	64	Root nodules
*P. mimosarum*	8.3	64	Root nodules
*P. oxyphila*	10.7	64	Soil
*P. sacchari*	7.3	64	Soil
*P. tropica*	8.6	65	Plant stem
*P. bannensis*	8.7	64	Plant roots
*C. humi*	7.6	63	Soil
*C. terrestris*	8.2	63	Soil
*C. choica*	9.8	63	Soil
*C. telluris*	7.1	64	Soil
‘*C. arationis*’	9.4	63	Soil
*C. glathei*	8.6	64	Soil
*C. sordidicola*	6.9	60	Fungus
*C. udeis*	10.1	60	Soil
‘*C. concitans*’	6.2	63	Clinical sample
*C. grimmiae*	6.7	63	Moss
*C. zhejiangensis*	7.8	63	Wastewater
‘*C. fortuita*’	7.4	63	Soil
‘*C. temeraria*’	8.3	63	Soil
*C. cordobensis*	8.2	64	Soil
‘*C. hypogeia*’	8.3	63	Soil
‘*C. calidae*’	9.6	63	Water
*C. megalochromosomata*	9.5	63	Soil
*C. jiangsuensis*	8.6	63	Soil
‘*C. pedi*’	9.1	63	Soil
‘*C. turbans*’	7.4	63	Clinical sample
‘*C. peredens*’	6.7	63	Soil
‘*C. ptereochthonis*’	7.7	64	Soil
‘*C. glebae*’	7.8	63	Soil
‘*C. arvi*’	9.7	62	Soil
‘*C. catudaia*’	7.7	63	Soil
*B. plantarii*	8.1	69	Soil
*B. glumae*	5.8	68	Plant leaves
*B. gladioli*	8.8	68	Plant roots
*B. mallei*	5.8	69	Clinical sample
*B. pseudomallei*	7.0	68	Clinical sample
*B. thailandensis*	6.4	68	Soil
*B. oklahomensis*	7.1	67	Clinical sample
*B. stagnalis*	7.5	68	Soil
*B. ubonensis*	6.9	67	Soil
*B. dolosa*	6.3	67	Clinical sample
*B. multivorans*	6.2	67	Clinical sample
*B. pseudomultivorans*	7.9	67	Clinical sample
*B. territorii*	6.9	66	Water
*B. diffusa*	6.9	66	Clinical sample
*B. latens*	6.5	67	Clinical sample
*B. vietnamiensis*	6.9	67	Soil
*B. ambifaria*	7.5	67	Soil
*B. pyrrocinia*	8.0	66	Soil
*B. stabilis*	8.0	66	Clinical sample
*B. cenocepacia*	8.1	67	Clinical sample
*B. anthina*	7.3	67	Soil
*B. seminalis*	7.6	67	Clinical sample
*B. cepacia*	8.6	67	Plant tissue
*B. contaminans*	9.3	66	Veterinary sample
*B. lata*	8.7	66	Soil

^a^*Species names in inverted commas (‘…’) have not yet been validly published*.

### Sequence Datasets and Multiple Alignments

A set of 106 genes with ≥95% complete sequences were identified among the genomes of 86 *Burkholderia* sensu lato species and the 6 outgroup taxa. The 106 genes were identified using a strict orthology estimation performed in EDGAR (Blom et al., 2016). Only those sequences with a mean % identity of 60.22 (median 54.63%) and a mean Expect(*E*)-value of 6.494625e-09 (median 1.00e-101) of the accepted BLAST hits were included in the final datasets. Although the full set of shared genes among these taxa would be considerably larger, the examined genomes differed substantially in their level of completeness and the annotation approaches utilized. Our conservative approach for generating these datasets therefore attempted to avoid inadvertently including phylogenetic noise caused by potential sequencing and annotation inconsistencies.

**FIGURE 1:**
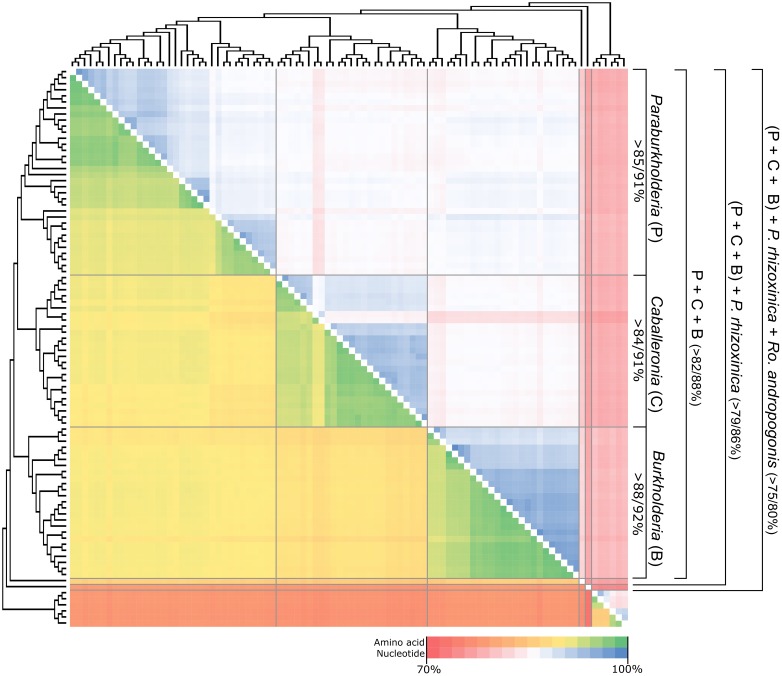
A heat map depicting the sequence similarity of the concatenated sequence of the conserved 106 genes used for phylogenetic analysis. The cladogram indicating the various intra- and intergeneric relationships were inferred from the amino acid based ML topology. Nucleotide similarity values are indicated in the upper triangle of the map, with amino acid similarity values indicated in the lower triangle of the map. A summary of the similarity values for the 5 lineages of interest are indicated for each group (nucleotide/amino acid %), in the panel on the right. For specific values, refer to Supplementary File S1.

The concatenated dataset for the 106 genes consisted of 92 taxa with 25499 residues in the amino acid alignment and 80027 bases in the nucleotide alignment. The amino acid dataset consisted of 99.1% coding characters with 0.9% of the dataset consisting of alignment gaps, while the nucleotide dataset consisted of 98.6% coding characters with 1.4% of the dataset consisting of alignment gaps. Neither of these datasets included any poorly aligned regions because of the absence of more divergent sequences. For example, the amino acid and nucleotide similarity across the entire dataset (including *Ralstonia* and *Cupriavidus* outgroups) were >77% and >73%, respectively (**Figure 1** and Supplementary File S1). Within each of the main phylogenetic clades inferred from the data (see below), these values were generally >92% and >84%, respectively.

Despite the high-level of conservation observed in the 106 genes, both the nucleotide and the amino acid data were free from significant levels of substitution saturation (Supplementary File S2). For both datasets, this was evident from the slope of the linear regression line for the plot between actual and modeled distances. However, compared to the nucleotide dataset, the amino acid dataset was least saturated, as the slope of its regression line was closest to 1. Our results also suggest the limited saturation present in the nucleotide data may be ascribed to multiple substitutions primarily occurring at third codon positions (Supplementary File S2).

We investigated the genomic distribution of the 106 genes by mapping them to those in the annotated genome of strain ATCC 25416^T^ of *Burkholderia cepacia*, which is also the type species for *Burkholderia* (Yabuuchi et al., 1992). These analyses showed that 101 of the genes mapped to chromosome 1 of this species (Supplementary Figure S1), where they appeared to be scattered throughout the replicon (see Supplementary Table S2 for the nucleotide positions and orientation of the respective genes). The remaining five genes mapped to chromosome 2 (Supplementary Figure S2 and Table S2).

Analysis of the putative functions of the 106 genes revealed that they are likely involved in a multitude of diverse functions. Based on both the original annotations for *B. cepacia* ATCC 25416^T^ and the KEGG analysis with GhostKOALA, only four of the 106 gene were classified as having unknown or hypothetical functions (Supplementary Table S3). About 44% of the remaining 102 genes represented “informational genes” (*sensu* Jain et al., 1999) and encoded products involved in processes relating to nucleotide synthesis, DNA replication and repair, transcription, translation and related processes. A further 35% of the genes encoded products involved in carbohydrate, lipid and amino acid metabolism, while the remaining 21% encoded products involved in diverse functions (e.g., signal transduction, membrane transport, iron scavenging, etc.) (Supplementary Table S3).

### Phylogenetic Analyses

Because of the limited substitution saturation detected in the concatenated amino acid and nucleotide datasets, both datasets were subjected to maximum likelihood phylogenetic analysis in RAxML “as is” (i.e., no attempt was made to exclude saturated sites). However, these analyses were conducted using substitution models specific for each gene, which in all cases accounted for invariable sites and included gammaa correction to account for among site rate variation. Although the nucleotide data partitions utilized the GTR model, each partition used independent model parameters (i.e., each gene partition utilized the six nucleotide substitution rates specific to it) (see Supplementary Table S2 for details on the substitution models used for the respective amino acid partitions).

**FIGURE 2:**
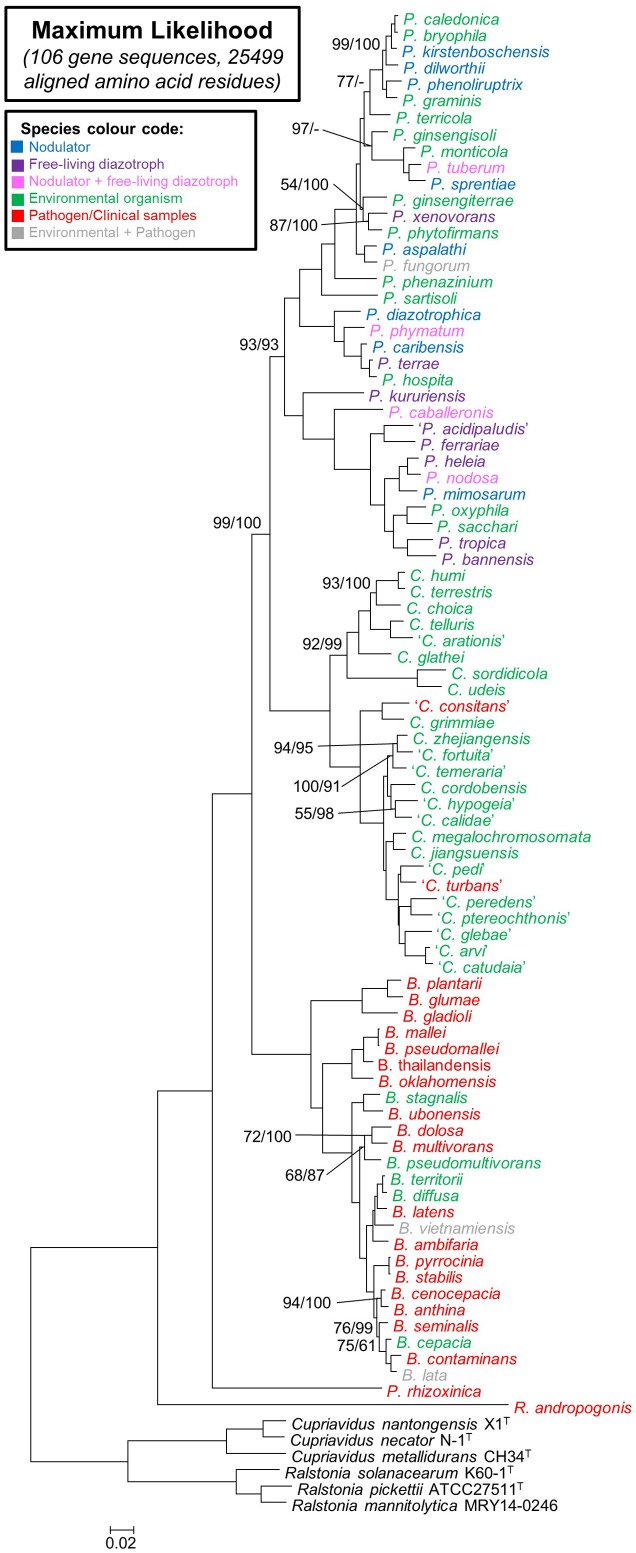
A maximum-likelihood phylogeny of the amino acid sequences of 106 concatenated genes for the 92 strains used in this study. A similar topology was obtained using the nucleotide sequences for these genes (Supplementary Figure S3). New combinations that have not yet been validated are indicated in inverted commas. General species substrates and origins are color coded according to the key provided. The majority of branches received 100% bootstrap in both the amino acid and nucleotide phylogenies and therefore only those branches in which 100% was not calculated for both analyses are indicated. Support is indicated in the order amino acid/nucleotide. The scale bar indicates the number of changes per site.

Highly similar and congruent topologies were inferred from the amino acid and nucleotide data for the 106 genes included in this study (**Figure 2** and Supplementary Figure S3). All of the branches in the two trees further received bootstrap support values exceeding 90% (with most supported by values of 100%). The only differences observed between the two trees were in terms of the placement of some species within certain terminal clades (e.g., in the nucleotide phylogeny *P. ginsengisoli* forms a distinct lineage within a larger clade containing *P. caledonica*, *P. bryophila*, *P. kirstenboschensis*, *P. dilworthii*, *P. phenoliruptrix*, *P. graminis*, *P. terricola*, *P. aspalathi*, *P. fungorum*, *P. ginsengiterrae*, *P. phytofirmans*, *P. xenovorans*, *P. monticola*, *P. tuberum*, and *P. sprentiae* but in the amino acid tree it is basal to a smaller clade consisting of *P. monticola*, *P. tuberum*, and *P. sprentiae*). These small topological differences probably reflect limited phylogenetic signal in the datasets for resolving more recent divergences. No disparities were observed regarding the composition of the main clades recovered from the two datasets.

In terms of the phylogenetic relationships among the taxa, both trees separated the *Burkholderia* sensu lato species into five distinct lineages (**Figure 1** and Supplementary Figure S3). Three of these corresponded to clades, respectively, representing *Paraburkholderia*, *Caballeronia*, and *Burkholderia* sensu stricto. The remaining two lineages were represented by *R. andropogonis* and *P. rhizoxinica*. Within this phylogeny, *Paraburkholderia* and *Caballeronia* were recovered as sister groups that shared an origin with *Burkholderia* sensu stricto. In turn, these three clades shared a most recent common ancestor with the lineage represented by *P. rhizoxinica*. Based on our analyses, the lineage represented by *R. andropogonis* is the most basal taxon in the *Burkholderia* sensu lato tree.

The *Paraburkholderia* clade consisted of 34 species. Of these, 33 were recently formally transferred to *Paraburkholderia* and the new combinations have been validated. Our data show that the novel combination (suggested by Sawana et al., 2014) requires *P. acidipaludis* still awaits validation. A similar situation exists for the *Caballeronia* clade. Of the 25 species it included, 12 were recently formally transferred to *Caballeronia*, but our results suggest that a further 13 (recently accepted as *Burkholderia* species) also need to be incorporated in this genus (**Table 3**).

**Table 3:** Summary of the novel combinations proposed for 13 species of *Caballeronia.*

New Combination	Basonym	Type Strain^a^	Reference
*Caballeronia arvi* comb. nov.	*Burkholderia arvi*	LMG 29317, CCUG68412, MAN34	Peeters et al., 2016
*Caballeronia arationis* comb. nov.	*Burkholderia arationis*	LMG 29324, CCUG 68405	Peeters et al., 2016
*Caballeronia calidae* comb. nov.	*Burkholderia calidae*	LMG 29321, CCUG 68408	Peeters et al., 2016
*Caballeronia catudaia* comb. nov.	*Burkholderia catudaia*	LMG 29318, CCUG 68411	Peeters et al., 2016
*Caballeronia concitans* comb. nov.	*Burkholderia concitans*	LMG 29315, CCUG 68414, AU12121	Peeters et al., 2016
*Caballeronia fortuita* comb. nov.	*Burkholderia fortuita*	LMG 29320, CCUG 68409	Peeters et al., 2016
*Caballeronia glebae* comb. nov.	*Burkholderia glebae*	LMG 29325, CCUG 68404	Peeters et al., 2016
*Caballeronia hypogeia* comb. nov.	*Burkholderia hypogeia*	LMG 29322, CCUG 68407	Peeters et al., 2016
*Caballeronia pedi* comb. nov.	*Burkholderia pedi*	LMG 29323, CCUG 68406	Peeters et al., 2016
*Caballeronia peredens* comb. nov.	*Burkholderia peredens*	LMG 29314, CCUG 68415, NF100	Peeters et al., 2016
*Caballeronia ptereochthonis* comb. nov.	*Burkholderia ptereochthonis*	LMG 29326, CCUG 68403	Peeters et al., 2016
*Caballeronia temeraria* comb. nov.	*Burkholderia temeraria*	LMG 29319, CCUG 68410	Peeters et al., 2016
*Caballeronia turbans* comb. nov.	*Burkholderia turbans*	LMG 29316, CCUG 68413, HI4065	Peeters et al., 2016

^a^*LMG = Belgian Coordinated Collections of Microorganisms, Laboratorium voor Microbiologie, Universiteit Gent; CCUG = Culture Collection, University of Göteborg, Department of Clinical Bacteriology, Institute of Clinical Bacteriology, Immunology, and Virology, University of Göteborg; The strain numbers starting with the abbreviations ‘MAN,’ ‘AU,’ ‘NF,’ and ‘HI’ are not part of international culture collections*.

## Discussion

To achieve our primary goal of resolving the generic boundaries and relationships within *Burkholderia* sensu lato, we endeavored to use as wide a taxon selection as possible. Therefore, to complement the genome data already in the public domain for 78 species in this assemblage, we determined the whole genome sequences for an additional eight PBE species. The genomes for these species exhibited similar characteristics as those of other members of *Burkholderia* sensu lato (see **Table 2**). This was particularly true in terms of genome size and total numbers of genes encoded. Some differences were observed in G+C content. As have been observed before (Gyaneshwar et al., 2011; Estrada-de los Santos et al., 2013; Sawana et al., 2014), the *Burkholderia* sensu stricto genomes were higher in G+C content than *Paraburkholderia* and *Caballeronia*, which were similar in G+C content. Future studies aimed at exploring genome architecture and the functions encoded on these genomes will undoubtedly reveal traits and processes that more clearly characterize the various lineages of this economically important assemblage of bacteria.

For inferring a robust phylogeny that are congruent with the evolutionary history of *Burkholderia* sensu lato, we attempted to avoid or limit the effect of factors known to negatively impact phylogenetic trees (Philippe et al., 2011). The criteria used for generating the respective datasets therefore focused on the use of orthologous loci and on limiting the effects of non-phylogenetic signal. The former was accomplished by using EDGAR to identify orthologous protein-coding genes (Blom et al., 2016). The orthologous nature of a large proportion of the genes included in our final dataset was also congruent with expectations of the so-called complexity hypothesis (Jain et al., 1999; Cohen et al., 2011). *In silico* functional analysis showed that about 44% of these genes represented “informational genes” with products that potentially participate in processes related to DNA replication and repair, transcription and translation. Due to the complexity of their interactions with different proteins and other cellular constituents, these genes are typically less prone to horizontal gene transfer (Jain et al., 1999; Cohen et al., 2011). Our approach for identifying suitable gene sequences from which to infer the phylogeny thus lessened the chances considerably of accidentally using paralogous or xenologous gene copies (Koonin, 2005).

To limit the amount of non-phylogenetic signal in the data, a three-tiered approach was used. [i] The final dataset was large, almost devoid of missing sites (i.e., where genes in some taxa were not sequenced in their entirety) and consisted of the sequences for 106 genes common to *Burkholderia* sensu lato and its *Ralstonia* and *Cupriavidus* outgroups. Such large datasets typically outperform smaller datasets that only contain the sequences for one or a few genes (Daubin et al., 2002; Coenye et al., 2005; Galtier and Daubin, 2008; Bennet et al., 2012; Chan et al., 2012). This is because the “true” phylogenetic signal inherent to orthologs included in such a large dataset will dominate the analysis and typically attenuate or dilute the effects of spurious non-phylogenetic signal associated with one or a few genes (Daubin et al., 2002; Andam and Gogarten, 2011). [ii] Lack of evolutionary independence among loci may contribute to non-phylogenetic signal during tree inference (Gevers et al., 2005). For example, genes that are clustered or whose products are involved in similar or linked processes typically experience similar evolutionary forces, which is accordingly also reflected in their phylogenies (i.e., these reflect the linked evolutionary history of the genes and not the evolutionary history of the species or genus). However, the 106 genes used for resolving *Burkholderia* sensu lato were not significantly clustered (see Supplementary Figures S1, S2), while their inferred products were predicted to participate in diverse functions (see Supplementary Table S3). [iii] Substitution saturation is another important source of non-phylogenetic signal (Philippe and Forterre, 1999; Xia et al., 2003; Jeffroy et al., 2006; Philippe et al., 2011), and to compensate for its limited occurrence in our datasets, all phylogenetic analyses utilized independent substitution models for each gene partition. This approach proved fairly successful as both the nucleotide and amino acid data supported congruent trees with highly similar topologies.

Our maximum likelihood analyses of the aligned amino acid and nucleotide sequences for 106 genes produced a highly supported phylogeny for *Burkholderia* sensu lato (see **Figure 2**). Most of the branches on this 92-taxon phylogeny received full (100%) bootstrap support. The generation of such a well-resolved phylogeny is, however, not unusual when large datasets containing the information of numerous genes are used. Various previous studies have shown the value of this approach for resolving systematic questions at taxonomic ranks from the genus level and up (e.g., Zhang et al., 2011; Richards et al., 2014; Ormeno-Orrillo et al., 2015; Rahman et al., 2015). Our study thus adds to the growing body of work demonstrating how genome-informed taxonomic decisions represent more robust solutions than those based solely on 16S rRNA or conventional MLSA.

Based on our results, boundaries can for the first time be confidently demarcated for *Burkholderia* sensu stricto, *Caballeronia* and *Paraburkholderia*. These three genera, respectively, represent three of the five distinct lineages recovered among the *Burkholderia* sensu lato species. *Burkholderia* sensu stricto is represented by a large clade that includes the *B. cepacia* complex as well as the *B. pseudomallei* group, and consists primarily of pathogenic species, as suggested previously (Gyaneshwar et al., 2011; Sawana et al., 2014; Estrada-de los Santos et al., 2016). The *Caballeronia* clade includes environmental species that initially formed part of Transition Group 2 of Estrada-de los Santos et al. (2016) and that were transferred to the genus *Caballeronia* by Dobritsa and Samadpour (2016). This clade also includes all 13 of the recently described and validated *Burkholderia glathei*-like species (Oren and Garrity, 2016; Peeters et al., 2016). Based on these findings, we propose the formal inclusion of these species in the genus *Caballeronia* (*sensu* Dobritsa and Samadpour, 2016) (see **Table 3** for details of the proposed new combinations). The inclusion of these taxa into *Caballeronia* raises the number of species to 25. Based on our analyses of their genomes, these species do not encode common *nod* or *nif* and *fix* loci, suggesting that none of the current *Caballeronia* species represent rhizobia or diazotrophs.

The *Paraburkholderia* clade is represented by diverse species, including both free-living and symbiotic diazotrophs, as well as environmental species. Although most of the taxa in this clade have already been formally transferred to *Paraburkholderia* (Sawana et al., 2014) and the novel combinations have been validated (Oren and Garrity, 2015a,b), this genus should also clearly include ‘*P. acidipaludis*’ (Aizawa et al., 2010) isolated from water chestnut as suggested by Sawana et al. (2014). This novel combination, however, still awaits validation. Interestingly, *Paraburkholderia* separates into two fully supported sub-clades, one including at least 23 species (spanning from *P. caledonica* to *P. hospita* in **Figure 2**) and the other including 11 species (*P. kururiensis* to *P. sacchari* in **Figure 2**). Although we could not identify any obvious reason for this split, future studies should explore its possible biological and taxonomic significance.

The two remaining lineages of *Burkholderia* sensu lato is represented by *R. andropogonis* [a pathogen of sorghum (Lopes-Santos et al., 2017)] and *P. rhizoxinica* [a member of Transition Group 1 of Estrada-de los Santos et al. (2016)]. Various previous studies have pointed out that these species should be excluded from *Burkholderia* sensu stricto, *Caballeronia* and/or *Paraburkholderia* (e.g., Estrada-de los Santos et al., 2013, 2016; Dobritsa and Samadpour, 2016). In fact, they have been suggested to represent new genera (Estrada-de los Santos et al., 2013; Dobritsa and Samadpour, 2016). This debate ultimately culminated in the introduction of the new genus *Robbsia* to accommodate *R. andropogonis* (Lopes-Santos et al., 2017). Based on our findings, the taxonomy of *P. rhizoxinica* requires similar revision. This species is definitely not a member of *Paraburkholderia* despite having been moved there from *Burkholderia* by Sawana et al. (2014). Both *R. andropogonis* and *P. rhizoxinica* currently represent the only members of their respective lineages for which whole genome sequences are available. Future studies should therefore seek to identify their respective congeneric species [some of which will likely include those in Transition Group 1 (Estrada-de los Santos et al., 2016)] and to understand the biological and evolutionary properties underlying these two lineages.

In addition to allowing unambiguous demarcation of the genera in *Burkholderia* sensu lato, this study also revealed, for the first time, the relationships among these taxa. *Burkholderia* sensu stricto has a well-supported sister group relationship with the clade containing *Caballeronia*, and *Paraburkholderia*. *P. rhizoxinica* is sister to the *Burkholderia* sensu stricto+*Caballeronia*+*Paraburkholderia* clade, while *R. andropogonis* occupies the most basal position in the tree. Knowledge about these relationships could inform hypotheses regarding the biology and evolution of these bacteria, especially in terms of virulence and pathogenicity. For example, *Burkholderia* sensu stricto primarily includes human and animal pathogens, while *P. rhizoxinica* and *Robbsia* are also represented by pathogens (Estrada-de los Santos et al., 2016; Lopes-Santos et al., 2017). Moreover, certain *Caballeronia* and *Paraburkholderia* species have also been isolated from clinical samples [e.g., ‘*C. consitans*’ and ‘*C. turbans*’ (Peeters et al., 2016) and *P. fungorum* (Coenye et al., 2001), and *P. tropica* (Deris et al., 2010), respectively]. The availability of a robust phylogenetic framework for these taxa would thus be invaluable for deciphering the processes and mechanisms involved in the evolution of these species.

## Description of New Species Combinations

### Description of *Caballeronia arvi* comb. nov.

*Caballeronia arvi* (ar’vi. L. gen. n. *arvi* of a field).

Basonym: *Burkholderia arvi* Peeters et al., 2016.

The description is as provided in Peeters et al. (2016). Analysis of 106 conserved protein-coding sequences have shown that this species is placed in the genus *Caballeronia* with very high support.

The type strain is LMG 29317^T^ (= CCUG 68412^T^ = MAN34^T^).

#### Description of *Caballeronia arationis* comb. nov.

*Caballeronia arationis* (a.ra.ti.o’nis. L. gen. n. *arationis* from a field).

Basonym: *Burkholderia arationis* Peeters et al., 2016.

The description is as provided in Peeters et al. (2016). Phylogenetic analysis of 106 conserved protein-coding loci clearly showed that there is high support for the placement of this species in *Caballeronia*.

The type strain is LMG 29324^T^ (=CCUG 68405^T^).

#### Description of *Caballeronia calidae* comb. nov.

*Caballeronia calidae* (ca’li.dae. L. gen. n. *calidae* from warm water, because this strain was isolated from pond water in a tropical garden).

Basonym: *Burkholderia calidae* Peeters et al., 2016.

The description is as provided in Peeters et al. (2016). Phylogenetic analysis of 106 conserved protein-coding loci showed (with a high degree of certainty) that this species belongs in the genus *Caballeronia*.

The type strain is LMG 29321^T^ (=CCUG 68408^T^).

#### Description of *Caballeronia catudaia* comb. nov.

*Caballeronia catudaia* (ca.tu.da’ia. Gr. adj. *catudaios* subterraneous; N. L. fem. adj. *catudaia*, earth-born).

Basonym: *Burkholderia catudaia* Peeters et al., 2016.

The description is as provided in Peeters et al. (2016). Our analyses of 106 conserved protein-coding loci clearly indicate that this species has high support for being included in *Caballeronia*.

The type strain is LMG 29318^T^ (=CCUG 68411^T^).

#### Description of *Caballeronia concitans* comb. nov.

*Caballeronia concitans* (con.ci’tans. L. fem. part. pres. *concitans* disturbing, upsetting; because the isolation of this bacterium from human sources, including blood, further disturbs the image of this lineage of *Burkholderia* species as benign bacteria).

Basonym: *Burkholderia concitans* Peeters et al., 2016.

The description is as provided in Peeters et al. (2016). Analysis of 106 conserved protein-coding loci showed that this species has high support for belonging to the genus *Caballeronia*.

The type strain is LMG 29315^T^ (=CCUG 68414^T^ = AU12121^T^).

#### Description of *Caballeronia fortuita* comb. nov.

*Caballeronia fortuita* (for.tu.i’ta. L. fem. adj. *fortuita* accidental, unpremeditated; referring to its fortuitous isolation when searching for *Burkholderia caledonica* endophytes).

Basonym: *Burkholderia fortuita* Peeters et al., 2016.

The description is as described in Peeters et al. (2016). Our analysis of 106 conserved protein-coding loci clearly show this species is included in the genus *Caballeronia*.

The type strain is LMG 29320^T^ (=CCUG 68409^T^).

#### Description of *Caballeronia glebae* comb. nov.

*Caballeronia glebae* (gle’bae. L. gen. n. *glebae* from a lump or clod of earth, soil).

Basonym: *Burkholderia glebae* Peeters et al., 2016.

The description appears in Peeters et al. (2016). Analysis of 106 conserved protein-coding loci shows high support for the placement of this species in the genus *Caballeronia*.

The type strain is LMG 29325^T^ (=CCUG 68404^T^).

#### Description of *Caballeronia hypogeia* comb. nov.

*Caballeronia hypogeia* (hy.po.ge’ia. Gr. adj. *hypogeios* subterraneous; N. L. fem. adj. *hypogeia*, subterraneous, earth-born).

Basonym: *Burkholderia hypogeia* Peeters et al., 2016.

The description appears in Peeters et al. (2016). Our analysis of 106 conserved protein-coding loci supports the inclusion of this species into the genus *Caballeronia*.

The type strain is LMG 29322^T^ (=CCUG 68407^T^).

#### Description of *Caballeronia pedi* comb. nov.

*Caballeronia pedi* (pe’di. Gr. n. *pedon* soil, earth; N. L. gen. n. *pedi*, from soil).

Basonym: *Burkholderia pedi* Peeters et al., 2016.

The description is listed in Peeters et al. (2016). The analysis of 106 conserved protein-coding loci, showed that this species is placed in *Caballeronia*.

The type strain is LMG 29323^T^ (=CCUG 68406^T^).

#### Description of *Caballeronia peredens* comb. nov.

*Caballeronia peredens* (per.e’dens. L. fem. part. pres. *peredens* consuming, devouring; referring to the capacity of this bacterium to degrade fenitrothion).

Basonym: *Burkholderia peredens* Peeters et al., 2016.

The description is as discussed in Peeters et al. (2016). Our analysis of 106 conserved protein-coding loci clearly shows that this species should be included in the genus *Caballeronia*.

The type strain is LMG 29314^T^ (=CCUG 68415^T^ = NF100^T^).

#### Description of *Caballeronia ptereochthonis* comb. nov.

*Caballeronia ptereochthonis* (pte.re.o.chtho’nis Gr. n. *pteris* fern; Gr. n. *chthon* soil; N. L. gen. n. *ptereochthonis*, from fern soil).

Basonym: *Burkholderia ptereochthonis* Peeters et al., 2016.

The description appears in Peeters et al. (2016). The analysis of 106 conserved protein-coding loci clearly shows that this species should be included in *Caballeronia*.

The type strain is LMG 29326^T^ (=CCUG 68403^T^).

#### Description of *Caballeronia temeraria* comb. nov.

*Caballeronia temeraria* (te.me.ra’ri.a. L. fem. adj. *temeraria* accidental, inconsiderate; referring to its accidental isolation when searching for *Burkholderia caledonica* endophytes).

Basonym: *Burkholderia temeraria* Peeters et al., 2016.

The description of this species appears in Peeters et al. (2016). The analysis of 106 conserved protein-coding loci here, shows that this species is included in *Caballeronia* with high support.

The type strain is LMG 29319^T^ (=CCUG 68410^T^).

#### Description of *Caballeronia turbans* comb. nov.

*Caballeronia turbans* (tur’bans. L. fem. part. pres. *turbans* disturbing, agitating, because the isolation of this bacterium from human pleural fluid further disturbs the image of this lineage of *Burkholderia* species as benign bacteria).

Basonym: *Burkholderia turbans* Peeters et al., 2016.

The original species description appears in Peeters et al. (2016). Our analysis of 106 conserved protein-coding loci shows that this species forms part of *Caballeronia*.

The type strain is LMG 29316^T^ (=CCUG 68413^T^ = HI4065^T^).

## Author Contributions

CB, MPa, PM, SV, and ES: Original concept; analyses; interpretation of results, writing and proofreading. WC, JA, and EvZ: Analyses, writing and proofreading. MH, AC, MPi, KP, NV, NM, DS, TR, CD, NS, VM, NI, NK, and TW: Genome analyses; interpretation of results; proofreading. JB and WW Genome analyses; interpretation of results; proofreading.

## Conflict of Interest Statement

The authors declare that the research was conducted in the absence of any commercial or financial relationships that could be construed as a potential conflict of interest.

## Footnotes

1 http://www.jgi.doe.gov

2 https://github.com/lh3/wgsim

3 https://edgar.computational.bio.uni-giessen.de

4 http://www.geneious.com

5 www.kegg.jp/blastkoala/

6 https://www.ncbi.nlm.nih.gov/
