# Corrigendum: Genome Data Provides High Support for Generic Boundaries in *Burkholderia* Sensu Lato

**DOI:** 10.3389/fmicb.2018.00373

**Published:** 2018-03-02

**Authors:** Chrizelle W. Beukes, Marike Palmer, Puseletso Manyaka, Wai Y. Chan, Juanita R. Avontuur, Elritha van Zyl, Marcel Huntemann, Alicia Clum, Manoj Pillay, Krishnaveni Palaniappan, Neha Varghese, Natalia Mikhailova, Dimitrios Stamatis, T. B. K. Reddy, Chris Daum, Nicole Shapiro, Victor Markowitz, Natalia Ivanova, Nikos Kyrpides, Tanja Woyke, Jochen Blom, William B. Whitman, Stephanus N. Venter, Emma T. Steenkamp

**Affiliations:** ^1^Department of Microbiology and Plant Pathology, Forestry and Agricultural Biotechnology Institute, University of Pretoria, Pretoria, South Africa; ^2^DOE Joint Genome Institute, Walnut Creek, CA, United States; ^3^Bioinformatics and Systems Biology, Justus-Liebig-University Giessen, Giessen, Germany; ^4^Department of Microbiology, University of Georgia, Athens, GA, United States

**Keywords:** *Burkholderia*, *Paraburkholderia*, *Caballeronia*, phylogenomics, *Robbsia andropogonis*, *Burkholderia rhizoxinica*

In the original article, there was a spelling mistake in a species name contained in Table 3 as published. As the information in this table is required for validation of the novel species combinations, this mistake has been corrected from “*Caballeronia ptereocthonis* comb. nov.” to “*Caballeronia ptereochthonis* comb. nov.” and “*Burkholderia ptereocthonis*” to “*Burkholderia ptereochthonis*”. The corrected Table 3 appears below.

**Table 3:** Summary of the novel combinations proposed for 13 species of *Caballeronia*.

**New Combination**	**Basonym**	**Type Strain^a^**	**Reference**
*Caballeronia arvi* comb. nov.	*Burkholderia arvi*	LMG 29317, CCUG68412, MAN34	Peeters et al., 2016
*Caballeronia arationis* comb. nov.	*Burkholderia arationis*	LMG 29324, CCUG 68405	Peeters et al., 2016
*Caballeronia calidae* comb. nov.	*Burkholderia calidae*	LMG 29321, CCUG 68408	Peeters et al., 2016
*Caballeronia catudaia* comb. nov.	*Burkholderia catudaia*	LMG 29318, CCUG 68411	Peeters et al., 2016
*Caballeronia concitans* comb. nov.	*Burkholderia concitans*	LMG 29315, CCUG 68414, AU12121	Peeters et al., 2016
*Caballeronia fortuita* comb. nov.	*Burkholderia fortuita*	LMG 29320, CCUG 68409	Peeters et al., 2016
*Caballeronia glebae* comb. nov.	*Burkholderia glebae*	LMG 29325, CCUG 68404	Peeters et al., 2016
*Caballeronia hypogeia* comb. nov.	*Burkholderia hypogeia*	LMG 29322, CCUG 68407	Peeters et al., 2016
*Caballeronia pedi* comb. nov.	*Burkholderia pedi*	LMG 29323, CCUG 68406	Peeters et al., 2016
*Caballeronia peredens* comb. nov.	*Burkholderia peredens*	LMG 29314, CCUG 68415, NF100	Peeters et al., 2016
*Caballeronia ptereochthonis* comb. nov.	*Burkholderia ptereochthonis*	LMG 29326, CCUG 68403	Peeters et al., 2016
*Caballeronia temeraria* comb. nov.	*Burkholderia temeraria*	LMG 29319, CCUG 68410	Peeters et al., 2016
*Caballeronia turbans* comb. nov.	*Burkholderia turbans*	LMG 29316, CCUG 68413, HI4065	Peeters et al., 2016

a *LMG = Belgian Coordinated Collections of Microorganisms, Laboratorium voor Microbiologie, Universiteit Gent; CCUG = Culture Collection, University of Göteborg, Department of Clinical Bacteriology, Institute of Clinical Bacteriology, Immunology, and Virology, University of Göteborg; The strain numbers starting with the abbreviations ‘MAN,’ ‘AU,’ ‘NF,’ and ‘HI’ are not part of international culture collections*.

Also, an additional section titled “Description of New Species Combinations” was added to the original article. The section contains a short protologue for each proposed novel combination to conform to the rules of the Bacterial Code of Nomenclature. The protologues appear below.

The authors apologize for this error. The original article has been updated.

## Description of new species combinations

### Description of *Caballeronia arvi* comb. nov.

*Caballeronia arvi* (ar'vi. L. gen. n. *arvi* of a field).

Basonym: *Burkholderia arvi* Peeters et al., 2016.

The description is as provided in Peeters et al. (2016). Analysis of 106 conserved protein-coding sequences have shown that this species is placed in the genus *Caballeronia* with very high support.

The type strain is LMG 29317^T^ (= CCUG 68412^T^ = MAN34^T^).

#### Description of *Caballeronia arationis* comb. nov.

*Caballeronia arationis* (a.ra.ti.o'nis. L. gen. n. *arationis* from a field).

Basonym: *Burkholderia arationis* Peeters et al., 2016.

The description is as provided in Peeters et al. (2016). Phylogenetic analysis of 106 conserved protein-coding loci clearly showed that there is high support for the placement of this species in *Caballeronia*.

The type strain is LMG 29324^T^ (=CCUG 68405^T^).

#### Description of *Caballeronia calidae* comb. nov.

*Caballeronia calidae* (ca'li.dae. L. gen. n. *calidae* from warm water, because this strain was isolated from pond water in a tropical garden).

Basonym: *Burkholderia calidae* Peeters et al., 2016.

The description is as provided in Peeters et al. (2016). Phylogenetic analysis of 106 conserved protein-coding loci showed (with a high degree of certainty) that this species belongs in the genus *Caballeronia*.

The type strain is LMG 29321^T^ (=CCUG 68408^T^).

#### Description of *Caballeronia catudaia* comb. nov.

*Caballeronia catudaia* (ca.tu.da'ia. Gr. adj. *catudaios* subterraneous; N. L. fem. adj. *catudaia*, earth-born).

Basonym: *Burkholderia catudaia* Peeters et al., 2016.

The description is as provided in Peeters et al. (2016). Our analyses of 106 conserved protein-coding loci clearly indicate that this species has high support for being included in *Caballeronia*.

The type strain is LMG 29318^T^ (=CCUG 68411^T^).

#### Description of *Caballeronia concitans* comb. nov.

*Caballeronia concitans* (con.ci'tans. L. fem. part. pres. *concitans* disturbing, upsetting; because the isolation of this bacterium from human sources, including blood, further disturbs the image of this lineage of *Burkholderia* species as benign bacteria).

Basonym: *Burkholderia concitans* Peeters et al., 2016.

The description is as provided in Peeters et al. (2016). Analysis of 106 conserved protein-coding loci showed that this species has high support for belonging to the genus *Caballeronia*.

The type strain is LMG 29315^T^ (=CCUG 68414^T^ = AU12121^T^).

#### Description of *Caballeronia fortuita* comb. nov.

*Caballeronia fortuita* (for.tu.i'ta. L. fem. adj. *fortuita* accidental, unpremeditated; referring to its fortuitous isolation when searching for *Burkholderia caledonica* endophytes).

Basonym: *Burkholderia fortuita* Peeters et al., 2016.

The description is as described in Peeters et al. (2016). Our analysis of 106 conserved protein-coding loci clearly show this species is included in the genus *Caballeronia*.

The type strain is LMG 29320^T^ (=CCUG 68409^T^).

#### Description of *Caballeronia glebae* comb. nov.

*Caballeronia glebae* (gle'bae. L. gen. n. *glebae* from a lump or clod of earth, soil).

Basonym: *Burkholderia glebae* Peeters et al., 2016.

The description appears in Peeters et al. (2016). Analysis of 106 conserved protein-coding loci shows high support for the placement of this species in the genus *Caballeronia*.

The type strain is LMG 29325^T^ (=CCUG 68404^T^).

#### Description of *Caballeronia hypogeia* comb. nov.

*Caballeronia hypogeia* (hy.po.ge'ia. Gr. adj. *hypogeios* subterraneous; N. L. fem. adj. *hypogeia*, subterraneous, earth-born).

Basonym: *Burkholderia hypogeia* Peeters et al., 2016.

The description appears in Peeters et al. (2016). Our analysis of 106 conserved protein-coding loci supports the inclusion of this species into the genus *Caballeronia*.

The type strain is LMG 29322^T^ (=CCUG 68407^T^).

#### Description of *Caballeronia pedi* comb. nov.

*Caballeronia pedi* (pe'di. Gr. n. *pedon* soil, earth; N. L. gen. n. *pedi*, from soil).

Basonym: *Burkholderia pedi* Peeters et al. 2016.

The description is listed in Peeters et al. (2016). The analysis of 106 conserved protein-coding loci, showed that this species is placed in *Caballeronia*.

The type strain is LMG 29323^T^ (=CCUG 68406^T^).

#### Description of *Caballeronia peredens* comb. nov.

*Caballeronia peredens* (per.e'dens. L. fem. part. pres. *peredens* consuming, devouring; referring to the capacity of this bacterium to degrade fenitrothion).

Basonym: *Burkholderia peredens* Peeters et al., 2016.

The description is as discussed in Peeters et al. (2016). Our analysis of 106 conserved protein-coding loci clearly shows that this species should be included in the genus *Caballeronia*.

The type strain is LMG 29314^T^ (=CCUG 68415^T^ = NF100^T^).

#### Description of *Caballeronia ptereochthonis* comb. nov.

*Caballeronia ptereochthonis* (pte.re.o.chtho'nis Gr. n. *pteris* fern; Gr. n. *chthon* soil; N. L. gen. n. *ptereochthonis*, from fern soil).

Basonym: *Burkholderia ptereochthonis* Peeters et al., 2016.

The description appears in Peeters et al. (2016). The analysis of 106 conserved protein-coding loci clearly shows that this species should be included in *Caballeronia*.

The type strain is LMG 29326^T^ (=CCUG 68403^T^).

#### Description of *Caballeronia temeraria* comb. nov.

*Caballeronia temeraria* (te.me.ra'ri.a. L. fem. adj. *temeraria* accidental, inconsiderate; referring to its accidental isolation when searching for *Burkholderia caledonica* endophytes).

Basonym: *Burkholderia temeraria* Peeters et al., 2016.

The description of this species appears in Peeters et al. (2016). The analysis of 106 conserved protein-coding loci here, shows that this species is included in *Caballeronia* with high support.

The type strain is LMG 29319^T^ (=CCUG 68410^T^).

#### Description of *Caballeronia turbans* comb. nov.

*Caballeronia turbans* (tur'bans. L. fem. part. pres. *turbans* disturbing, agitating, because the isolation of this bacterium from human pleural fluid further disturbs the image of this lineage of *Burkholderia* species as benign bacteria).

Basonym: *Burkholderia turbans* Peeters et al., 2016.

The original species description appears in Peeters et al. (2016). Our analysis of 106 conserved protein-coding loci shows that this species forms part of *Caballeronia*.

The type strain is LMG 29316^T^ (=CCUG 68413^T^ = HI4065^T^).

#### Conflict of interest statement

The authors declare that the research was conducted in the absence of any commercial or financial relationships that could be construed as a potential conflict of interest.
