# Supplementary material for: Genome Data Provides High Support for Generic Boundaries in Burkholderia Sensu Lato
Source: Front Microbiol. 2017 Jun 26;8:1154. doi: 10.3389/fmicb.2017.01154 (PMC5483467; doi:10.3389/fmicb.2017.01154)
Supplement: Supplementary file 1 [file Table_1.PDF]

Supplementary Table S1. Details regarding the genomes for the 86 *Burkholderia* sensu lato species included in this study.

Species Name[a]	Genome characteristics[b]			NCBI Accession Number	NCBI BioProject
	Size (Mb)	G+C (%)	Number of genes		
Burkholderia sensu stricto					
B. ambifaria AMMD[T] = LMG 19182[T]	7.5	66.8	6580	GCA_000203915.1	PRJNA240122
B. anthina AZ-4-2-10-S1-D7	7.3	67.2	6401	GCA_001547525.1	PRJNA279182
B. cenocepacia J2315[T] = LMG 16656	8.1	66.9	7275	GCA_000009485.1	PRJNA339
B. cepacia ATCC 25416[T]	8.6	66.6	7682	GCA_001411495.1	PRJNA298860
B. contaminans LMG 23361[T]	9.3	65.9	8294	GCA_000987075.1	PRJNA203156
B. diffusa MSMB377	6.9	66.4	6157	GCA_001531975.1	PRJNA279182
B. dolosa PC543	6.3	67.1	5611	GCA_000497165.1	PRJNA214965
B. gladioli NBRC 13700[T]	8.8	67.7	7450	GCA_000739755.1	PRJDB252
B. glumae LMG 2196[T]	5.8	67.5	5749	GCA_000300755.1	PRJNA160241
B. lata 383[T] = LMG 22485[T]	8.7	66.3	7695	GCA_000012945.1	PRJNA10695
B. latens RF32-BP12	6.5	66.5	5767	GCA_001525985.1	PRJNA279182

**Species Name**[a]	**Genome characteristics**[b]			**NCBI Accession Number**	**NCBI BioProject**
	**Size (Mb)**	**G+C (%)**	**Number of genes**		
B. mallei ATCC 23344^T	5.8	68.5	5506	GCA_000011705.1	PRJNA171
B. multivorans ATCC BAA-247^T	6.2	67.1	5843	GCA_000286555.1	PRJNA62777
B. oklahomensis C6786^T = LMG 23618^T	7.1	67.1	6115	GCA_001522135.1	PRJNA279182
B. plantarii ATCC 43733^T	8.1	68.6	6666	GCA_001411805.1	PRJNA237833
B. pseudomallei ATCC 23343^T	7.0	68.3	6803*	GCA_001182285.1	PRJEB2196
B. pseudomultivorans SUB-INT24-BP10	7.9	67.4	6999	GCA_001525825.1	PRJNA279182
B. pyrrocinia DSM 10685^T	8.0	66.5	7029	GCA_001028665.1	PRJNA283474
B. seminalis FL-5-5-10-S1-D0	7.6	67.4	6749	GCA_001524085.1	PRJNA279182
B. stabilis LA20W	8.0	66.4	6957	GCA_001685505.1	PRJDB4654
B. stagnalis MSMB1638	7.5	67.6	6620	GCA_001526815.1	PRJNA279182
B. territorii MSMB2203	6.9	66.4	6046	GCA_001636095.1	PRJNA279182
B. thailandensis E264^T = CCUG 48851^T	6.4	67.6	5475	GCA_000152285.1	PRJNA19251

Species Name[a]	Genome characteristics[b]			NCBI Accession Number	NCBI BioProject
	Size (Mb)	G+C (%)	Number of genes		
B. ubonensis Bu	6.9	67.3	6733	GCA_000170335.1	PRJNA19539
B. vietnamiensis LMG 10929^T	6.9	66.8	6015	GCA_000959445.1	PRJNA235223
Caballeronia					
'*C. arationis*' LMG 29324^T	9.4	62.8	8857*	GCA_001544975.1	PRJEB12493
'*C. arvi*' LMG 29317^T	9.7	62.4	9055*	GCA_001544695.1	PRJEB12485
'*C. calidae*' LMG 29321^T	9.6	62.5	8631	GCA_900044055.2	PRJEB12490
'*C. catudaia*' LMG 29318^T	7.7	62.8	7346*	GCA_001544755.1	PRJEB12486
C. choica LMG 22940^T	9.8	62.6	9302*	GCA_001544535.1	PRJEB12479
'*C. concitans*' LMG 29315^T	6.2	63.2	5835*	GCA_001544615.1	PRJEB12483
C. cordobensis LMG 27620^T	8.2	63.7	7712*	GCA_001544575.1	PRJEB12481
'*C. fortuita*' LMG 29320^T	7.4	62.9	6819	GCA_001544835.2	PRJEB12489
C. glathei DSM 50014^T	8.6	64.4	7808	GCA_000698595.1	PRJNA238428

**Species Name**[a]	**Genome characteristics**[b]			**NCBI Accession Number**	**NCBI BioProject**
	**Size (Mb)**	**G+C (%)**	**Number of genes**		
'*C. glebae*' LMG 29325^T	7.8	62.7	7316*	GCA_001545035.1	PRJEB12494
C. grimmiae R27^T = DSM 25160^T	6.7	63.0	6204	GCA_000698555.1	PRJNA238424
C. humi LMG 22934^T	7.6	62.8	7126*	GCA_001544475.1	PRJEB12476
'*C. hypogeia*' LMG 29322^T	8.3	63.2	7601	GCA_001544875.2	PRJEB12491
C. jiangsuensis MP-1^T = LMG 27927^T	8.6	62.6	7876	GCA_000648925.1	PRJNA238425
C. megalochromosomata JC2949^T = JCM 19905^T	9.5	62.7	8584	GCA_001580565.1	PRJNA241423
'*C. pedi*' LMG 29323^T	9.1	63.0	8578*	GCA_001544915.1	PRJEB12492
'*C. peredens*' LMG 29314^T	6.7	63.1	6175*	GCA_001544595.1	PRJEB12482
'*C. ptereochthonis*' LMG 29326^T	7.7	64.2	7298*	GCA_001545075.1	PRJEB12495
C. sordidicola LMG 22029^T	6.9	60.1	6126	GCA_001544455.2	PRJEB12475
C. telluris LMG 22936^T	7.1	64.0	6588*	GCA_001544495.1	PRJEB12477
'*C. temeraria*' LMG 29319^T	8.3	62.7	7757	GCA_001544795.2	PRJEB12488

Species Name[a]	Genome characteristics[b]			NCBI Accession Number	NCBI BioProject
	Size (Mb)	G+C (%)	Number of genes		
C. terrestris LMG 22937[T]	8.2	62.6	7752*	GCA_001544515.1	PRJEB12478
'*C. turbans*' LMG 29316[T]	7.4	63.1	7117*	GCA_001544655.1	PRJEB12484
C. udeis LMG 27134[T]	10.1	60.0	9093	GCA_001544555.2	PRJEB12480
C. zhejiangensis OP-1[T] = KTCT 23300[T]	7.8	62.7	7232	GCA_000698575.1	PRJNA238427
Paraburkholderia					
'*P. acidipaludis*' NBRC 101816[T]	6.5	65.2	5793	GCA_000684975.1	PRJDB247
P. andropogonis Ba3549	6.2	58.9	5414	GCA_000566705.1	PRJNA228914
'*P. aspalathi*' LMG 27731[T]	9.9	61.1	9038	GCA_900116445.1 (This study)	PRJNA323251
P. bannensis NBRC 103871[T]	8.7	64.0	7603	GCA_000685015.1	PRJDB248
P. bryophila 376MFSha3.1	7.4	61.9	6536	GCA_000383275.1	PRJNA187936
'*P. caballeronis*' LMG 26416[T]	7.1	67.0	6258	GCA_900109675.1 (This study)	PRJNA323249
P. caledonica NBRC 102488[T]	7.3	62.0	6513	GCA_000685095.1	PRJDB288

Species Name[a]	Genome characteristics[b]			NCBI Accession Number	NCBI BioProject
	Size (Mb)	G+C (%)	Number of genes		
P. caribensis MWAP64^T = LMG 18531^T	9.0	62.6	7894	GCA_001449005.1	PRJNA294731
P. diazotrophica LMG 26031^T	8.7	62.6	8041	GCA_900108945.1 (This study)	PRJNA323247
P. dilworthii WSM3556^T = LMG 27173^T	7.7	61.8	6664	GCA_000472525.1	PRJNA182743
P. ferrariae NBRC 106233^T	7.9	64.8	7100	GCA_000685035.1	PRJDB249
P. fungorum NBRC 102489^T	8.7	61.8	7857	GCA_000685055.1	PRJDB250
P. ginsengisoli NBRC 100965^T	6.5	63.6	5756	GCA_000739735.1	PRJDB251
P. ginsengiterrae DCY85^T = JCM 19888^T	8.5	62.5	7719	GCA_001645125.1	PRJNA319663
P. graminis C4D1M^T = LMG 18924^T	7.5	62.9	6617	GCA_000172415.1	PRJNA20537
P. heleia NBRC 101817^T	8.0	64.6	7174	GCA_000739775.1	PRJDB253
P. hospita LMG 20598^T	11.2	61.9	10534	GCA_900108355.1 (This study)	PRJNA323250
P. kirstenboschensis Kb15^T = LMG 28727^T	8.3	61.8	8738*	GCA_001636975.1	PRJNA190073
P. kururiensis JCM 10599^T	6.8	64.2	6551*	GCA_000616025.1	PRJDB655

**Species Name**[a]	**Genome characteristics**[b]			**NCBI Accession Number**	**NCBI BioProject**
	**Size (Mb)**	**G+C (%)**	**Number of genes**		
P. mimosarum LMG 23256[T]	8.3	63.9	7422	GCA_000472825.1	PRJNA163559
P. monticola JC2948[T] = JCM 19904[T]	7.9	63.8	7003	GCA_001580545.1	PRJNA240348
P. nodosa DSM21604[#]	9.5	64.1	8522	GCA_000519185.1	PRJNA165365
P. oxyphila NBRC 105797[T]	10.7	64.1	9394	GCA_000685075.1	PRJDB256
P. phenazinium LMG 2247[T]	8.6	62.3	7927	GCA_900100735.1 (This study)	PRJNA323253
P. phenoliruptrix AC1100[T] = LMG 22037[T]	7.8	63.1	7233	GCA_000416445.1	PRJNA205406
P. phymatum STM815[T] = LMG 21445[T]	8.7	62.2	7703	GCA_000020045.1	PRJNA17409
P. phytofirmans PsJN[T] = CCUG 49060[T]	8.2	62.3	7313	GCA_000020125.1	PRJNA17463
P. rhizoxinica HKI454[T] = DSM 19002[T]	3.8	60.7	3209	GCA_000198775.1	PRJEA51915
P. sacchari LMG 19450[T]	7.3	64.0	6443	GCA_000785435.1	PRJNA263694
P. sartisoli LMG 24000[T]	5.9	63.5	5407	GCA_900107685.1 (This study)	PRJNA323254
P. sprentiae WSM5005[T] = LMG 27175[T]	7.8	63.2	7174*	GCA_000473465.1	PRJNA66661

**Species Name**[a]	**Genome characteristics**[b]			**NCBI Accession Number**	**NCBI BioProject**
	**Size (Mb)**	**G+C (%)**	**Number of genes**		
P. terrae NBRC 100964^T	9.9	62.0	8801	GCA_000739835.1	PRJDB257
P. terricola LMG 20594^T	7.3	63.6	6748	GCA_900142195.1 (This study)	PRJNA323256
P. tropica LMG 22274^T	8.6	64.8	7611	GCA_900109265.1 (This study)	PRJNA323257
P. tuberum STM678^T = LMG 21444^T	9.0	63.0	8658	PRJNA51167	PRJNA51167
P. xenovorans LB400^T = LMG 21463^T	9.7	62.6	8623	GCA_000013645.1	PRJNA254

[a] LMG = BCCM/LMG = Belgian Co-ordinated collections of micro-organisms/Laboratorium voor Microbiologie

Address: Laboratory of Microbiology, Department of Biochemistry and Microbiology, Faculty of Sciences of Ghent University, Ghent, Belgium

ATCC = American Type Culture Collection.

Address: ATCC, 10801 University Boulevard, Manassa, VA 20110-2209, USA

NBRC = Biological Resource Center

Address: Biological Resource Center, National Institute of Technology and Evaluation (NITE), 2-5-8, Kazusakamatari, Kisarazu-shi, Chiba Pref., 2920-0812, Japan

DSM = DSMZ = DSMZ-Deutsche Sammlung von Mikroorganismen und Zellkulturen

Address: DSMZ-Deutsche Sammlung von Mikroorganismen und Zellkulturen GmbH, Inhoffenstrasse 7B, D-38124 Braunschweig, Germany.

CCUG = Culture Collection, University of Göteberg

Address: Culture Collection, University of Göteborg, Department of Clinical Bacteriology, Institute of Clinical, Bacteriology, Immunology, and Virology, Guldhedsgatn 10A s-413, 46 Göteborg, Sweden

JCM = Japan Collection of Microorganisms

Address: Japan Collection of Microorganisms, RIKEN BioResource Center, Tsukuba, Ibaraki 305-0074, Japan

KCTC = Korean Collection for Type Cultures

Address: Korean Collection for Type Cultures, Korea Research Institute of Bioscience & Biotechnology, Yusong, Taejon 305-600, Republic of Korea.

Those strains which do not have superscript "T" are not type strains and are therefore not normally deposited at a culture collection.

[#] This is the type strain for *P. nodosa* according to NCBI.

[b] The asterisk in this column indicate those strains for which annotation was determined by RAST (Rapid Annotations using Subsystems Technology; http://rast.nmpdr.org
