# Supplementary material for: Genome Data Provides High Support for Generic Boundaries in Burkholderia Sensu Lato
Source: Front Microbiol. 2017 Jun 26;8:1154. doi: 10.3389/fmicb.2017.01154 (PMC5483467; doi:10.3389/fmicb.2017.01154)
Supplement: Supplementary file 2 [file Table_2.PDF]

Supplementary Table S2. Chromosome positions and model information of 106 genes included in the concatenated datasets.

Gene number	Chromosomal location, direction and products of genes [a]				Substitution model and sequence similarity [b]	
	**Original annotations**	**Chromosome number**	**Position on chromosome**	**Direction**	**Amino Acid Model of Evolution**	**% Protein similarity**
4	50S ribosomal protein L1	1	2,749,638 - 2,750,336	reverse	Dayhoff	77.4% - 100%
5	50S ribosomal protein L10	1	2,748,845 - 2,749,342	reverse	LG	76.7% - 100%
7	30S ribosomal protein S12	1	2,736,881 - 2,737,261	reverse	DCMut	91.2% - 100%
8	30S ribosomal protein S7	1	2,736,238 - 2,736,708	reverse	LG	81.7% - 100%
9	50S ribosomal protein L14	1	2,725,326 - 2,725,694	reverse	LG	85.6% - 100%
11	30S ribosomal protein S8	1	2,723,729 - 2,724,124	reverse	LG	73.6% - 100%
13	50S ribosomal protein L18	1	2,722,802 - 2,723,167	reverse	WAG	69.5% - 100%
14	30S ribosomal protein S5	1	2,722,269 - 2,722,787	reverse	Dayhoff	70.3% - 100%
15	50S ribosomal protein L30	1	2,722,071 - 2,722,253	reverse	LG	83.3% - 100%
16	50S ribosomal protein L15	1	2,721,610 - 2,722,044	reverse	LG	74.1% - 100%
18	30S ribosomal protein S13	1	2,719,450 - 2,719,815	reverse	LG	72.7% - 100%
20	30S ribosomal protein S4	1	2,718,242 - 2,718,865	reverse	LG	80.2% - 100%
21	DNA-directed RNA polymerase subunit alpha	1	2,717,140 - 2,718,117	reverse	JTT	86.5% - 100%

Gene number	Chromosomal location, direction and products of genes [a]				Substitution model and sequence similarity [b]	
	**Original annotations**	**Chromosome number**	**Position on chromosome**	**Direction**	**Amino Acid Model of Evolution**	**% Protein similarity**
30	glutamate synthase	1	2,682,090 - 2,683,556	reverse	Dayhoff	68.0% - 100%
43	1-(5-phosphoribosyl)-5-((5-phosphoribosylamino)methylideneamino)imidazole-4-carboxamide isomerase	1	2,652,942 - 2,653,697	reverse	WAG	81.6% - 100%
55	glutathione S-transferase	1	2,642,831 - 2,643,442	reverse	LG	85.1% - 100%
61	monothiol glutaredoxin, Grx4 family	1	2,586,279 - 2,586,590	forward	Dayhoff	73.3% - 100%
63	peptide chain release factor 1	1	2,584,279 - 2,585,361	forward	JTT	73.3% - 100%
70	glutamine amidotransferase	1	2,561,293 - 2,561,883	forward	WAG	73.0% - 100%
73	Co2+/Mg2+ efflux protein ApaG	1	2,557,420 - 2,557,794	reverse	Dayhoff	61.3% - 100%
76	phospho-N-acetylmuramoyl-pentapeptide- transferase	1	2,518,539 - 2,519,708	reverse	LG	76.3% - 100%
79	UDP-3-O-[3-hydroxymyristoyl] N-acetylglucosamine deacetylase	1	2,507,085 - 2,508,002	reverse	LG	74.4% - 100%
86	50S ribosomal protein L27	1	2,491,960 - 2,492,223	reverse	WAG	76.2% - 100%

Gene number	Chromosomal location, direction and products of genes [a]				Substitution model and sequence similarity [b]	
	**Original annotations**	**Chromosome number**	**Position on chromosome**	**Direction**	**Amino Acid Model of Evolution**	**% Protein similarity**
87	glutamate 5-kinase	1	2,489,450 - 2,490,568	reverse	JTT	76.1% - 100%
88	proline--tRNA ligase	1	2,486,116 - 2,487,852	reverse	LG	70.1% - 100%
94	acetyl-CoA carboxylase biotin carboxylase subunit	1	2,469,390 - 2,470,757	forward	LG	85.4% - 100%
103	transcriptional repressor	1	2,419,782 - 2,420,210	forward	Dayhoff	66.9% - 100%
116	bifunctional phosphoribosylaminoimidazolecarboxamide formyltransferase/inosine monophosphate cyclohydrolase	1	43,318 - 44,502	reverse	Dayhoff	78.4% - 100%
117	Fis family transcriptional regulator	1	2,371,551 - 2,371,784	forward	LG	55.8% - 100%
123	preprotein translocase subunit SecF	1	2,341,246 - 2,342,196	forward	LG	74.6% - 100%
132	phospho-2-dehydro-3-deoxyheptonate aldolase	1	2,316,146 - 2,317,219	forward	LG	76.9% - 100%
154	NrdR family transcriptional regulator	1	2,215,894 - 2,216,373	reverse	LG	61.2% - 100%
156	molecular chaperone GroES	1	2,135,773 - 2,136,066	reverse	RtREV	84.2% - 100%
172	6,7-dimethyl-8-ribityllumazine synthase	1	2,046,476 - 2,046,994	forward	LG	70.6% - 100%
180	cell division topological specificity factor	1	2,016,072 - 2,016,326	reverse	LG	73.5% - 100%

Gene number	Chromosomal location, direction and products of genes [a]				Substitution model and sequence similarity [b]	
	**Original annotations**	**Chromosome number**	**Position on chromosome**	**Direction**	**Amino Acid Model of Evolution**	**% Protein similarity**
185	DNA-directed RNA polymerase subunit omega	1	2,004,039 - 2,004,242	reverse	LG	75.0% - 100%
203	lipopolysaccharide assembly protein LapB	1	1,942,464 - 1,943,636	reverse	JTT	53.9% - 100%
205	ADP-L-glycero-D-mannoheptose-6-epimerase	1	1,938,959 - 1,939,951	reverse	JTT	74.7% - 100%
206	cysteine synthase B	1	1,937,421 - 1,938,323	reverse	LG	77.7% - 100%
209	methionine ABC transporter permease	1	1,932,190 - 1,932,843	reverse	LG	74.2% - 100%
224	ferredoxin	1	1,894,972 - 1,895,295	forward	Dayhoff	76.5% - 100%
230	50S ribosomal protein L32	1	1,875,041 - 1,875,220	reverse	Dayhoff	79.7% - 100%
235	acyl carrier protein	1	1,870,629 - 1,870,868	reverse	LG	87.3% - 100%
237	RNA polymerase sigma factor RpoE	1	1,868,061 - 1,868,660	reverse	LG	76.9% - 100%
244	elongation factor P	1	1,853,757 - 1,854,314	forward	JTT	62.8% - 100%
245	CDP-diacylglycerol--glycerol-3-phosphate 3-phosphatidyltransferase	1	1,849,656 - 1,850,243	reverse	JTT	65.6% - 100%
249	carbamoyl-phosphate synthase small subunit	1	1,683,838 - 1,684,983	reverse	WAG	76.7% - 100%

Gene number	Chromosomal location, direction and products of genes [a]				Substitution model and sequence similarity [b]	
	**Original annotations**	**Chromosome number**	**Position on chromosome**	**Direction**	**Amino Acid Model of Evolution**	**% Protein similarity**
258	DNA-binding response regulator	1	1,667,105 - 1,667,806	reverse	JTT	76.5% - 100%
267	succinyl-CoA--3-ketoacid-coenzyme A transferase subunit A	1	1,441,079 - 1,441,783	reverse	LG	70.3% - 100%
272	50S ribosomal protein L35	1	1,431,237 - 1,431,434	reverse	Dayhoff	71.0% - 100%
310	nucleoside-diphosphate kinase	1	859,756 - 860,181	forward	LG	81.6% - 100%
317	hypothetical protein	1	3,792,930 - 3,793,124	reverse	LG	58.3% - 100%
318	thiol reductase thioredoxin	1	845,957 - 846,283	reverse	LG	73.1% - 100%
334	inorganic phosphate transporter	1	802,828 - 803,838	forward	LG	74.4% - 100%
336	50S ribosomal protein L9	1	800,082 - 800,534	forward	LG	64.9% - 100%
341	hypothetical protein	1	849,360 - 849,686	forward	WAG	62.5% - 100%
346	intracellular septation protein A	1	761,630 - 762,160	reverse	JTT	63.7% - 100%
352	endopeptidase La	1	742,256 - 744,679	forward	JTT	86.0% - 100%
364	ubiquinone-binding protein	1	660,510 - 660,947	forward	LG	64.8% - 100%
377	ribosome recycling factor	1	641,554 - 642,114	forward	JTT	75.8% - 100%

Gene number	Chromosomal location, direction and products of genes [a]				Substitution model and sequence similarity [b]	
	**Original annotations**	**Chromosome number**	**Position on chromosome**	**Direction**	**Amino Acid Model of Evolution**	**% Protein similarity**
379	30S ribosomal protein S2	1	638,801 - 639,541	forward	LG	76.8% - 100%
412	3-deoxy-8-phosphooctulonate synthase	1	492,488 - 493,342	forward	WAG	77.4% - 100%
413	CTP synthetase	1	490,833 - 492,491	forward	WAG	79.3% - 100%
419	ferredoxin, 2Fe-2S type, ISC system	1	476,765 - 477,106	forward	Dayhoff	66.4% - 100%
423	iron-sulfur cluster scaffold-like protein	1	473,335 - 473,742	forward	Dayhoff	82.5% - 100%
427	phasin protein	1	464,740 - 465,306	reverse	JTT	41.1% - 100%
440	fumarate hydratase	1	369,554 - 371,077	reverse	WAG	80.4% - 100%
442	biopolymer transporter	1	365,878 - 366,609	reverse	LG	61.8% - 100%
443	biopolymer transporter ExbD	1	365,436 - 365,864	reverse	WAG	44.9% - 100%
454	NADH-quinone oxidoreductase subunit L	1	328,667 - 330,721	forward	LG	79.3% - 100%
455	NADH-quinone oxidoreductase subunit K	1	328,344 - 328,649	forward	LG	78.6% - 100%
457	NADH-quinone oxidoreductase subunit H	1	326,005 - 327,072	forward	LG	70.1% - 100%

Gene number	Chromosomal location, direction and products of genes [a]				Substitution model and sequence similarity [b]	
	**Original annotations**	**Chromosome number**	**Position on chromosome**	**Direction**	**Amino Acid Model of Evolution**	**% Protein similarity**
458	NADH-quinone oxidoreductase subunit F	1	322,281 - 323,591	forward	LG	83.3% - 100%
459	NADH-quinone oxidoreductase subunit D	1	320,360 - 321,613	forward	LG	85.4% - 100%
460	NADH-quinone oxidoreductase subunit C	1	319,748 - 320,350	forward	JTT	68.0% - 100%
462	NADH-quinone oxidoreductase subunit A	1	318,825 - 319,184	forward	LG	72.3% - 100%
474	transcriptional regulator	1	268,133 - 268,861	reverse	JTT	76.9% - 100%
475	phosphoribosylamine--glycine ligase	1	266,751 - 268,028	reverse	JTT	71.1% - 100%
489	nitrogen regulatory protein P-II 1	1	3,453,915 - 3,454,253	forward	Dayhoff	81.3% - 100%
491	inorganic pyrophosphatase	1	144,015 - 144,542	forward	Dayhoff	73.7% - 100%
492	lysine decarboxylase	1	123,268 - 125,547	forward	JTT	72.2% - 100%
493	deoxycytidine triphosphate deaminase	1	122,617 - 123,186	forward	JTT	87.8% - 100%
505	CysB family transcriptional regulator	1	76,740 - 77,681	reverse	LG	67.4% - 100%
507	acetylornithine transaminase purH	1	2,371,829 - 2,373,394	forward	WAG	74.4% - 100%

Gene number	Chromosomal location, direction and products of genes [a]				Substitution model and sequence similarity [b]	
	**Original annotations**	**Chromosome number**	**Position on chromosome**	**Direction**	**Amino Acid Model of Evolution**	**% Protein similarity**
524	isocitrate dehydrogenase (NADP(+))	1	6,383 - 7,639	forward	LG	70.7% - 100%
527	hypothetical protein	1	782,428 - 783,207	forward	JTT	75.0% - 100%
529	adenylate kinase	1	3,791,147 - 3,791,809	reverse	WAG	71.9% - 100%
535	nucleotide-binding protein	1	3,783,462 - 3,783,947	reverse	LG	78.3% - 100%
556	DNA recombination/repair protein RecA	1	3,687,428 - 3,688,498	forward	LG	85.3% - 100%
571	TetR family transcriptional regulator	1	3,613,429 - 3,614,028	forward	JTT	70.8% - 100%
575	ubiquinone/menaquinone biosynthesis C-methyltransferase UbiE	1	3,603,451 - 3,604,182	forward	LG	75.3% - 100%
585	ribosome hibernation promoting factor	1	3,537,440 - 3,537,799	reverse	LG	62.0% - 100%
586	HPr kinase/phosphorylase	1	3,535,632 - 3,536,600	reverse	JTT	77.2% - 100%
593	50S ribosomal protein L25	1	3,526,563 - 3,527,168	reverse	LG	57.8% - 100%
607	phosphocarrier protein HPr	1	3,459,096 - 3,459,365	forward	WAG	66.3% - 100%
610	nitrogen regulatory protein P-II 1	1	152,123 - 152,461	forward	LG	81.3% - 100%

Gene number	Chromosomal location, direction and products of genes [a]				Substitution model and sequence similarity [b]	
	**Original annotations**	**Chromosome number**	**Position on chromosome**	**Direction**	**Amino Acid Model of Evolution**	**% Protein similarity**
637	S-adenosylmethionine synthase	1	3,202,047 - 3,203,234	forward	LG	81.4% - 100%
644	exodeoxyribonuclease III	1	3,175,692 - 3,176,465	forward	WAG	67.2% - 100%
652	7-carboxy-7-deazaguanine synthase	1	3,161,607 - 3,162,239	forward	Dayhoff	63.5% - 100%
655	protease HtpX homolog	1	3,154,141 - 3,154,998	forward	LG	82.7% - 100%
670	transamidase GatB domain protein	2	2,296,020 - 2,296,466	forward	Dayhoff	63.2% - 100%
672	aconitate hydratase	2	92,770 - 95,355	forward	WAG	79.9% - 100%
674	malate dehydrogenase	2	2,349,229 - 2,350,215	reverse	WAG	74.1% - 100%
677	3-isopropylmalate dehydratase large subunit	2	2,369,177 - 2,370,586	forward	LG	79.1% - 100%
678	3-isopropylmalate dehydratase small subunit	2	2,370,805 - 2,371,455	forward	WAG	74.4% - 100%
685	acetyl-CoA carboxylase subunit beta	2	2,382,912 - 2,383,784	forward	LG	82.7% - 100%

[a] Original Annotations, Chromosome number, Position on Chromosome and Direction all refer to the results of the comparison of our chosen genes to the annotated genome of *Burkholderia cepacia* ATCC 25416^T.

[b] Models of evolution: Dayhoff (Dayhoff et al., 1978); LG (Le and Gascuel, 2008); DCMut (Kosiol and Goldman, 2005); WAG (Whelan and Goldman, 2001); JTT (Jones, Taylor and Thornton, 1992); RtREV (Dimmic et al., 2002)

Dayhoff, M.O., Schwartz, R.M., and Orcutt, B.C. (1978). A Model of Evolutionary Change in Proteins. Atlas of protein sequence and structure. Vol. 5. *National Biomedical Research Foundation Silver Spring*, MD. 345-352.

Le, S.Q., Gascuel, O. (2008). An improved general amino acid replacement matrix. *Molecular Biology and Evolution* 25: 1307-1320.

Kosiol, C., Goldman, N. (2005). Different versions of the Dayhoff rate matrix. *Molecular Biology and Evolution* 22: 193-199.

Whelan, S., Goldman, N. (2001). A general empirical model of protein evolution derived from multiple protein families using a maximum-likelihood approach. *Molecular Biology and Evolution*, 18: 691-699.

Jones, D.T., Taylor, W.R., Thornton, J.M. (1992). The rapid generation of mutation data matrices from protein sequences. *Computer Applications in the Biosciences: CABIOS* 8: 275-282.

Dimmic, M.W., Rest, J.S., Mindell, D.P., Goldstein, R.A. (2002). rtREV: an amino acid substitution matrix for inference of retrovirus and reverse transcriptase phylogeny. *Journal of Molecular Evolution* 55: 65-73.
