# Supplementary material for: Genome Data Provides High Support for Generic Boundaries in Burkholderia Sensu Lato
Source: Front Microbiol. 2017 Jun 26;8:1154. doi: 10.3389/fmicb.2017.01154 (PMC5483467; doi:10.3389/fmicb.2017.01154)
Supplement: Supplementary file 3 [file Table_3.PDF]

Supplementary Table S3. Functional information for the proteins encoded by the 106 genes utilized for inferring the phylogeny of *Burkholderia* sensu lato.

Gene no.	Original Annotations[a]	Kyoto Encyclopedia of Genes and Genomes (KEGG) [b]			
		Class	Process/Function	Gene name	Protein/gene abbreviation
1	50S ribosomal protein L1	Translation	Ribosome	Large subunit ribosomal protein L1	RP-L1, MRPL1, rplA
2	50S ribosomal protein L10	Translation	Ribosome	Large subunit ribosomal protein L10	rplJ
3	30S ribosomal protein S12	Translation	Ribosome	Small subunit ribosomal protein S12	RPn-S12, MRPS12, rpsL
4	30S ribosomal protein S7	Translation	Ribosome	Small subunit ribosomal protein S7	RP-S7, MRPS7, rpsG
5	50S ribosomal protein L14	Translation	Ribosome	Large subunit ribosomal protein L14	RP-L14, MRPL14, rplN
6	30S ribosomal protein S8	Translation	Ribosome	Small subunit ribosomal protein S8	RP-S8, rpsH
7	50S ribosomal protein L18	Translation	Ribosome	Large subunit ribosomal protein L18	RP-L18, MRPL18, rplR
8	30S ribosomal protein S5	Translation	Ribosome	Small subunit ribosomal protein S5	RP-S5, MRPS5, rpsE
9	50S ribosomal protein L30	Translation	Ribosome	Large subunit ribosomal protein L30	RP-L30, MRPL30, rpmD
10	50S ribosomal protein L15	Translation	Ribosome	Large subunit ribosomal protein L15	RP-L15, MRPL15, rplO
11	30S ribosomal protein S13	Translation	Ribosome	Small subunit ribosomal protein S13	RP-S13, rpsM
12	30S ribosomal protein S4	Translation	Ribosome	Small subunit ribosomal protein S4	RP-S4, rpsD
13	DNA-directed RNA polymerase subunit alpha	Nucleotide metabolism	Purine metabolism	DNA-directed RNA polymerase subunit alpha	rpoA
			Pyrimidine metabolism	DNA-directed RNA polymerase subunit alpha	rpoA
		Transcription	RNA polymerase	DNA-directed RNA polymerase subunit alpha	rpoA
14	Glutamate synthase	Energy metabolism	Nitrogen metabolism	Glutamate synthase (NADPH/NADH) small chain	gltD
		Amino acid metabolism	Alanine, aspartate and glutamate metabolism	Glutamate synthase (NADPH/NADH) small chain	gltD
15	1-(5-phosphoribosyl)-5-((5-phosphoribosylamino)methylideneamino)imidazole-4-carboxamide isomerase	Amino acid metabolism	Histidine metabolism	Phosphoribosylformimino-5-aminoimidazole carboxamide ribotide isomerase	hisA
16	Glutathione S-transferase	Unclassified	Unknown	Hypothetical	NA

Gene no.	Original Annotations[a]	Kyoto Encyclopedia of Genes and Genomes (KEGG) [b]			
		Class	Process/Function	Gene name	Protein/gene abbreviation
17	Monothiol glutaredoxin, Grx4 family	Energy metabolism	Oxidative phosphorylation	NADH-quinone oxidoreductase subunit F	nuoF
18	Peptide chain release factor 1	Unclassified	Unknown	Hypothetical	NA
19	Glutamine amidotransferase	Amino acid metabolism	Phenylalanine, tyrosine and tryptophan biosynthesis	Anthranilate synthase component II	trpG
		Cellular community	Quorum sensing	Anthranilate synthase component II	trpG
20	Co2+/Mg2+ efflux protein ApaG	Unclassified	Unknown	Hypothetical	NA
21	Phospho-N-acetylmuramoyl-pentapeptide-transferase	Glycan biosynthesis and metabolism	Peptidoglycan biosynthesis	Phospho-N-acetylmuramoyl-pentapeptide-transferase	mraY
22	UDP-3-O-[3-hydroxymyristoyl] N-acetylglucosamine deacetylase	Glycan biosynthesis and metabolism	Lipopolysaccharide biosynthesis	UDP-3-O-[3-hydroxymyristoyl] N-acetylglucosamine deacetylase	lpxC
23	50S ribosomal protein L27	Translation	Ribosome	Large subunit ribosomal protein L27	RP-L27, MRPL27, rpmA
24	Glutamate 5-kinase	Amino acid metabolism	Arginine and proline metabolism	Glutamate 5-kinase	ProB
		Biosynthesis of other secondary metabolites	Carbapenem biosynthesis	Glutamate 5-kinase	proB
25	Proline-tRNA ligase	Translation	Aminoacyl-tRNA biosynthesis	Prolyl-tRNA synthetase	PARS, proS

Gene no.	Original Annotations[a]	Kyoto Encyclopedia of Genes and Genomes (KEGG) [b]			
		Class	Process/Function	Gene name	Protein/gene abbreviation
26	Acetyl-CoA carboxylase biotin carboxylase subunit	Carbohydrate metabolism	Pyruvate metabolism	Acetyl-CoA carboxylase, biotin carboxylase subunit	accC
			Propanoate metabolism	Acetyl-CoA carboxylase, biotin carboxylase subunit	accC
		Energy metabolism	Carbon fixation pathways in prokaryotes	Acetyl-CoA carboxylase, biotin carboxylase subunit	accC
		Lipid metabolism	Fatty acid biosynthesis	Acetyl-CoA carboxylase, biotin carboxylase subunit	accC
		Metabolism of terpenoids and polyketides	Tetracycline biosynthesis	Acetyl-CoA carboxylase, biotin carboxylase subunit	accC
27	Transcriptional repressor	Unclassified	Unknown	Hypothetical	NA
28	Bifunctional phosphoribosylaminoimidazolecarboxamide formyltransferase/inosine monophosphate cyclohydrolase	Nucleotide metabolism	Purine metabolism	Phosphoribosylaminoimidazolecarboxamide formyltransferase / IMP cyclohydrolase	PurH
		Metabolism of cofactors and vitamins	One carbon pool by folate	Phosphoribosylaminoimidazolecarboxamide formyltransferase / IMP cyclohydrolase	purH
29	Fis family transcriptional regulator	Unclassified	Unknown	Hypothetical	NA
30	Preprotein translocase subunit SecF	Folding, sorting and degradation	Protein export	Preprotein translocase subunit SecF	secF
		Membrane transport	Bacterial secretion system	Preprotein translocase subunit SecF	secF

Gene no.	Original Annotations[a]		Kyoto Encyclopedia of Genes and Genomes (KEGG) [b]		
		Class	Process/Function	Gene name	Protein/gene abbreviation
31	Phospho-2-dehydro-3-deoxyheptonate aldolase	Amino acid metabolism	Phenylalanine, tyrosine and tryptophan biosynthesis	3-deoxy-7-phosphoheptulonate synthase	aroF, aroG, aroH
		Cellular community	Quorum sensing	3-deoxy-7-phosphoheptulonate synthase	aroF, aroG, aroH
32	NrdR family transcriptional regulator	Unclassified	Unknown	Hypothetical	NA
33	Molecular chaperone GroES	Unclassified	Unknown	Hypothetical	NA
34	6,7-dimethyl-8-ribityllumazine synthase	Metabolism of cofactors and vitamins	Riboflavin metabolism	6,7-dimethyl-8-ribityllumazine synthase	ribH, RIB4
35	Cell division topological specificity factor	Unclassified	Unknown	Hypothetical	NA
36	DNA-directed RNA polymerase subunit omega	Nucleotide metabolism	Purine metabolism	DNA-directed RNA polymerase subunit omega	rpoZ
			Pyrimidine metabolism	DNA-directed RNA polymerase subunit omega	rpoZ
		Transcription	RNA polymerase	DNA-directed RNA polymerase subunit omega	rpoZ
37	Lipopolysaccharide assembly protein LapB	Unclassified	Unknown	Hypothetical	NA
38	ADP-L-glycero-D-mannoheptose-6-epimerase	Glycan biosynthesis and metabolism	Lipopolysaccharide biosynthesis	ADP-L-glycero-D-manno-heptose 6-epimerase	gmhD, rfaD
39	Cysteine synthase B	Energy metabolism	Sulfur metabolism	Cysteine synthase B	cysM
		Amino acid metabolism	Cysteine and methionine metabolism	cysteine synthase B	cysM

Gene no.	Original Annotations[a]		Kyoto Encyclopedia of Genes and Genomes (KEGG) [b]		
		Class	Process/Function	Gene name	Protein/gene abbreviation
40	Methionine ABC transporter permease	Membrane transport	ABC transporters	Preprotein translocase subunit SecF	secF
41	Ferredoxin	Unclassified	Unknown	Hypothetical	NA
42	50S ribosomal protein L32	Translation	Ribosome	Large subunit ribosomal protein L32	RP-L32, MRPL32, rpmF
43	Acyl carrier protein	Unclassified	Unknown	Hypothetical	NA
44	RNA polymerase sigma factore RpoE	Unclassified	Unknown	Hypothetical	NA
45	Elongation factor P	Unclassified	Unknown	Hypothetical	NA
46	CDP-diacylglycerol--glycerol-3-phosphate 3-phosphatidyltransferase	Lipid metabolism	Glycerophospholipid	Cardiolipin synthase	CRLS
47	Carbamoyal-phosphate synthase small subunit	Nucleotide metabolism	Pyrimidine metabolism	Carbamoyl-phosphate synthase small subunit	carA, CPA1
		Amino acid metabolism	Alanine, aspartate and glutamate metabolism	Carbamoyl-phosphate synthase small subunit	carA, CPA1
48	DNA-binding response regulator	Signal transduction	Two-component system	Two-component system, OmpR family, phosphate regulon response regulator PhoB	phoB
49	Succinyl-Co--3-ketoacid-coenzyme A transferase subunit A	Carbohydrate metabolism	Butanoate metabolism	3-oxoacid CoA-transferase subunit A	scoA
		Lipid metabolism	Synthesis and degradation of ketone bodies	3-oxoacid CoA-transferase subunit A	scoA
		Amino acid metabolism	Valine, leucine and isoleucine degradation	3-oxoacid CoA-transferase subunit A	scoA
50	50S ribosomal protein L35	Translation	Ribosome	Large subunit ribosomal protein L35	RP-L35, MRPL35, rpmI
51	Nucleoside-diphosphate kinase	Nucleotide metabolism	Purine metabolism	Nucleoside-diphosphate kinase	ndk, NME
			Pyrimidine metabolism	Nucleoside-diphosphate kinase	ndk, NME

Gene no.	Original Annotations[a]		Kyoto Encyclopedia of Genes and Genomes (KEGG) [b]		
		Class	Process/Function	Gene name	Protein/gene abbreviation
52	Hypothetical protein	Unclassified	Unknown	Hypothetical	NA
53	Thiol reductase thioredoxin	Unclassified	Unknown	Hypothetical	NA
54	Inorganic phosphate transporter	Unclassified	Unknown	Hypothetical	NA
55	50S ribosomal protein L9	Translation	Ribosome	Large subunit ribosomal protein L9	RP-L9, MRPL9, rplI
56	Hypothetical protein	Unclassified	Unknown	Hypothetical	NA
57	Intracellular septation protein A	Unclassified	Unknown	Hypothetical	NA
58	Endopeptidase La	Cell growth and death	Cell cycle	ATP-dependent Lon protease	lon
59	Ubiquinone-binding protein	Unclassified	Unknown	Hypothetical	NA
60	Ribosome recycling factor	Unclassified	Unknown	Hypothetical	NA
61	30S ribosomal protein S2	Translation	Ribosome	Small subunit ribosomal protein S2	RP-S2, MRPS2, rpsB
62	3-deoxy-8-phosphooctulonate synthase	Glycan biosynthesis and metabolism	Lipopolysaccharide biosynthesis	2-dehydro-3-deoxyphosphooctonate aldolase (KDO 8naP synthase)	kdsA
63	CTP synthetase	Nucleotide metabolism	Pyrimidine metabolism	CTP synthase	pyrG, CTPS
64	Ferredoxin, 2Fe-2S type, ISC system	Unclassified	Unknown	Hypothetical	NA
65	Iron-sulfur cluster scaffold-like protein	Unclassified	Unknown	Hypothetical	NA
66	Phasin protein	Unclassified	Unknown	Hypothetical	NA
67	Fumarate hydratase	Carbohydrate metabolism	Citric acid cycle	Fumarate hydratase, class I	fumA, fumB
			Pyruvate metabolism	Fumarate hydratase, class I	fumA, fumB
		Energy metabolism	Carbon fixation pathways in prokaryotes	Fumarate hydratase, class I	fumA, fumB
68	Biopolymer transporter	Unclassified	Unknown	Hypothetical	NA

Gene no.	Original Annotations[a]		Kyoto Encyclopedia of Genes and Genomes (KEGG) [b]		
		Class	Process/Function	Gene name	Protein/gene abbreviation
69	Biopolymer transporter ExbD	Unclassified	Unknown	Hypothetical	NA
70	NADH-quinone oxidoreductase subunit L	Energy metabolism	Oxidative phosphorylation	NADH-quinone oxidoreductase subunit L	nuoL
71	NADH-quinone oxidoreductase subunit K	Energy metabolism	Oxidative phosphorylation	NADH-quinone oxidoreductase subunit K	nuoK
72	NADH-quinone oxidoreductase subunit H	Energy metabolism	Oxidative phosphorylation	NADH-quinone oxidoreductase subunit H	nuoH
73	NADH-quinone oxidoreductase subunit F	Energy metabolism	Oxidative phosphorylation	NADH-quinone oxidoreductase subunit F	nuoF
74	NADH-quinone oxidoreductase subunit D	Energy metabolism	Oxidative phosphorylation	NADH-quinone oxidoreductase subunit D	nuoD
75	NADH-quinone oxidoreductase subunit C	Energy metabolism	Oxidative phosphorylation	NADH-quinone oxidoreductase subunit C	nuoC
76	NADH-quinone oxidoreductase subunit A	Energy metabolism	Oxidative phosphorylation	NADH-quinone oxidoreductase subunit A	nuoA
77	Transcriptional regulator	Unclassified	Unknown	Hypothetical	NA
78	Phosphoribosylamine--glycine ligase	Nucleotide metabolism	Purine metabolism	Phosphoribosylamine--glycine ligase	purD
79	Nitrogen regulatory protein P-II 1	Signal transduction	Two-component system	Nitrogen regulatory protein P-II 1	glnB
80	Inorganic pyrophosphatase	Energy metabolism	Oxidative phosphorylation	Inorganic pyrophosphatase	ppa
81	Lysine decarboxylase	Amino acid metabolism	Arginine and proline metabolism	Arginine decarboxylase	adiA
82	Deoxycytidine triphosphate deaminase	Nucleotide metabolism	Pyrimidine metabolism	dCTP deaminase	dcd
83	CysB family transcriptional regulator	Unclassified	Unknown	Hypothetical	NA

Gene no.	Original Annotations[a]	Kyoto Encyclopedia of Genes and Genomes (KEGG) [b]			
		Class	Process/Function	Gene name	Protein/gene abbreviation
84	Acetylornithine transaminase purH	Amino acid metabolism	Lysine biosynthesis	Acetylornithine/N-succinyldiaminopimelate aminotransferase	argD
			Arginine biosynthesis	Acetylornithine/N-succinyldiaminopimelate aminotransferase	argD
85	Isocitrate dehydrogenase (NADP(+))	Carbohydrate metabolism	Citric acid cycle	Isocitrate dehydrogenase	Icd
		Energy metabolism	Carbon fixation pathways in prokaryotes	Isocitrate dehydrogenase	IDH1, IDH2, icd
		Metabolism of other amino acids	Glutathione metabolism	Isocitrate dehydrogenase	IDH1, IDH2, icd
		Transport and catabolism	Peroxisome	Isocitrate dehydrogenase	IDH1, IDH2, icd
86	Hypothetical protein	Unclassified	Unknown	Hypothetical	NA
87	Adenylate kinase	Nucleotide metabolism	Purine metabolism	Adenylate kinase	adk, AK
88	Nucleotide-binding protein	Unclassified	Unknown	Hypothetical	NA
89	DNA recombination/repair protein RecA	Replication and repair	Homologous recombination	Recombination protein RecA	recA
90	TetR family transcriptional regulator	Unclassified	Unknown	Hypothetical	NA
91	Ubiquinone/menaquinone biosynthesis C-methyltransferase UbiE	Metabolism of cofactors and vitamins	Ubiquinone and other terpenoid-quinone biosynthesis	Demethylmenaquinone methyltransferase / 2-methoxy-6-polyprenyl-1,4-benzoquinol methylase	ubiE
92	Ribosome hibernation promoting factor	Unclassified	Unknown	Hypothetical	NA
93	HPr kinase/phosphorylase	Unclassified	Unknown	Hypothetical	NA
94	50S ribosomal protein L25	Translation	Ribosome	Large subunit ribosomal protein L25	RP-L25, rplY
95	Phosphocarrier protein HPr	Unclassified	Unknown	Hypothetical	NA

Gene no.	Original Annotations[a]		Kyoto Encyclopedia of Genes and Genomes (KEGG) [b]		
		Class	Process/Function	Gene name	Protein/gene abbreviation
96	Nitrogen regulatory protein P-II 1	Unclassified	Unknown	Hypothetical	NA
97	S-adenosylmethionine	Amino acid metabolism	Cysteine and methionine metabolism	S-adenosylmethionine synthetase	metK
98	Exodeoxyribonuclease III	Replication and repair	Base excision repair	Exodeoxyribonuclease III	xthA
99	7-carboxy-7-deazaguanine synthase	Unclassified	Unknown	Hypothetical	NA
100	Protease HtpX homolog	Unclassified	Unknown	Hypothetical	NA
101	Transamidase GatB domain protein	Unclassified	Unknown	Hypothetical	NA
102	Aconitate hydratase	Carbohydrate metabolism	Citric acid cycle	Aconitate hydratase	ACO, acnA
		Energy metabolism	Glyoxylate and dicarboxylate metabolism	Aconitate hydratase	ACO, acnA
			Carbon fixation pathways in prokaryotes	Aconitate hydratase	ACO, acnA

Gene no.	Original Annotations[a]		Kyoto Encyclopedia of Genes and Genomes (KEGG) [b]		
		Class	Process/Function	Gene name	Protein/gene abbreviation
103	Malate dehydrogenase	Carbohydrate metabolism	Citric acid cycle	Malate dehydrogenase	mdh
		Carbohydrate metabolism	Pyruvate metabolism	Malate dehydrogenase	mdh
		Carbohydrate metabolism	Glyoxylate and dicarboxylate metabolism	Malate dehydrogenase	mdh
		Energy metabolism	Carbon fixation in photosynthetic organisms	Malate dehydrogenase	mdh
		Energy metabolism	Carbon fixation pathways in prokaryotes	Malate dehydrogenase	mdh
		Energy metabolism	Methane metabolism	Malate dehydrogenase	mdh
		Amino acid metabolism	Cysteine and methionine metabolism	Malate dehydrogenase	mdh
104	3-isopropylmalate dehydratase large subunit	Carbohydrate metabolism	C5-Branched dibasic acid metabolism	3-isopropylmalate/(R)-2-methylmalate dehydratase large subunit	leuC
		Amino acid metabolism	Valine, leucine and isoleucine biosynthesis	3-isopropylmalate/(R)-2-methylmalate dehydratase large subunit	leuC
105	3-isopropylmalate dehydratase small subunit	Carbohydrate metabolism	C5-Branched dibasic acid metabolism	3-isopropylmalate/(R)-2-methylmalate dehydratase small subunit	leuD
		Amino acid metabolism	Valine, leucine and isoleucine biosynthesis	3-isopropylmalate/(R)-2-methylmalate dehydratase small subunit	leuD

Gene no.	Original Annotations[a]		Kyoto Encyclopedia of Genes and Genomes (KEGG) [b]		
		Class	Process/Function	Gene name	Protein/gene abbreviation
106	Acetyl-CoA carboxylase subunit beta	Carbohydrate metabolism	Pyuvate metabolism	Acetyl-CoA carboxylase carboxyl transferase subunit beta	accD
		Carbohydrate metabolism	Propanoate metabolism	Acetyl-CoA carboxylase carboxyl transferase subunit beta	accD
		Energy metabolism	Carbon fixation pathways in prokaryote	Acetyl-CoA carboxylase carboxyl transferase subunit beta	accD
		Lipid metabolism	Fatty acid biosynthesis	Acetyl-CoA carboxylase carboxyl transferase subunit beta	accD
		Metabolism of terpenoids and polyketides	Tetracycline biosynthesis	Acetyl-CoA carboxylase carboxyl transferase subunit beta	accD

[a]The column "Original Annotations" refers to the results of the comparison of the specific gene to the annotated genome of *Burkholderia cepacia* ATCC 25416[T].

[b] Inferred using the KEGG pathways database (http://www.genome.jp/kegg/pathway.html) and GhostKOALA (Kanehisa et al 2016). NA = Not Applicable.
