# Supplementary material for: Genome Data Provides High Support for Generic Boundaries in Burkholderia Sensu Lato
Source: Front Microbiol. 2017 Jun 26;8:1154. doi: 10.3389/fmicb.2017.01154 (PMC5483467; doi:10.3389/fmicb.2017.01154)
Supplement: Supplementary file 6 [file Image_1.PDF]

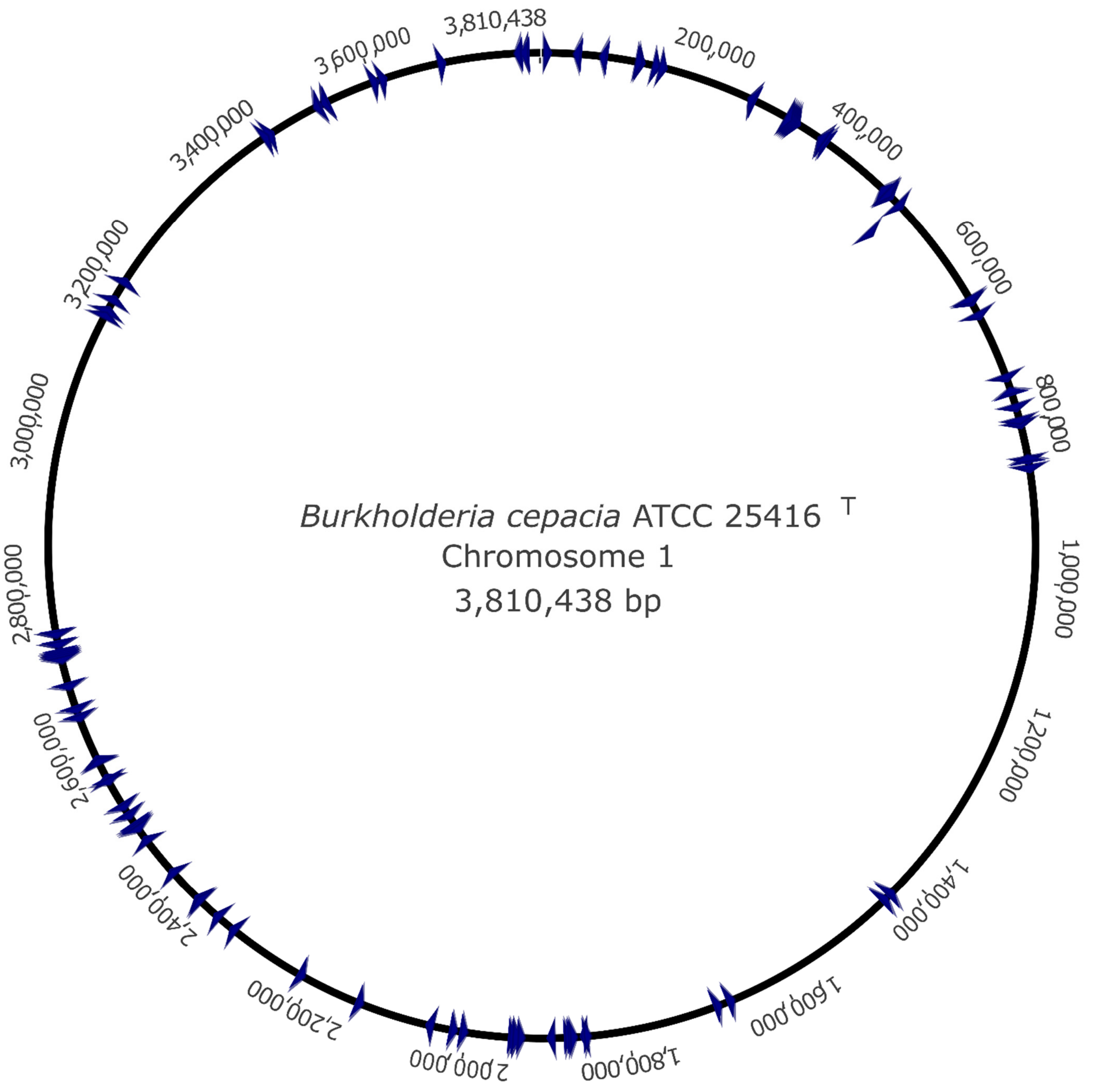

Supplementary Figure S1. The chromosomal positions of the 101 conserved genes present on chromosome 1, using *B. cepacia* ATCC 25416 [T] as reference. For specific positions, gene names and additional information, please refer to Supplementary Table S1.
