# Supplementary material for: Genome Data Provides High Support for Generic Boundaries in Burkholderia Sensu Lato
Source: Front Microbiol. 2017 Jun 26;8:1154. doi: 10.3389/fmicb.2017.01154 (PMC5483467; doi:10.3389/fmicb.2017.01154)
Supplement: Supplementary file 7 [file Image_2.PDF]

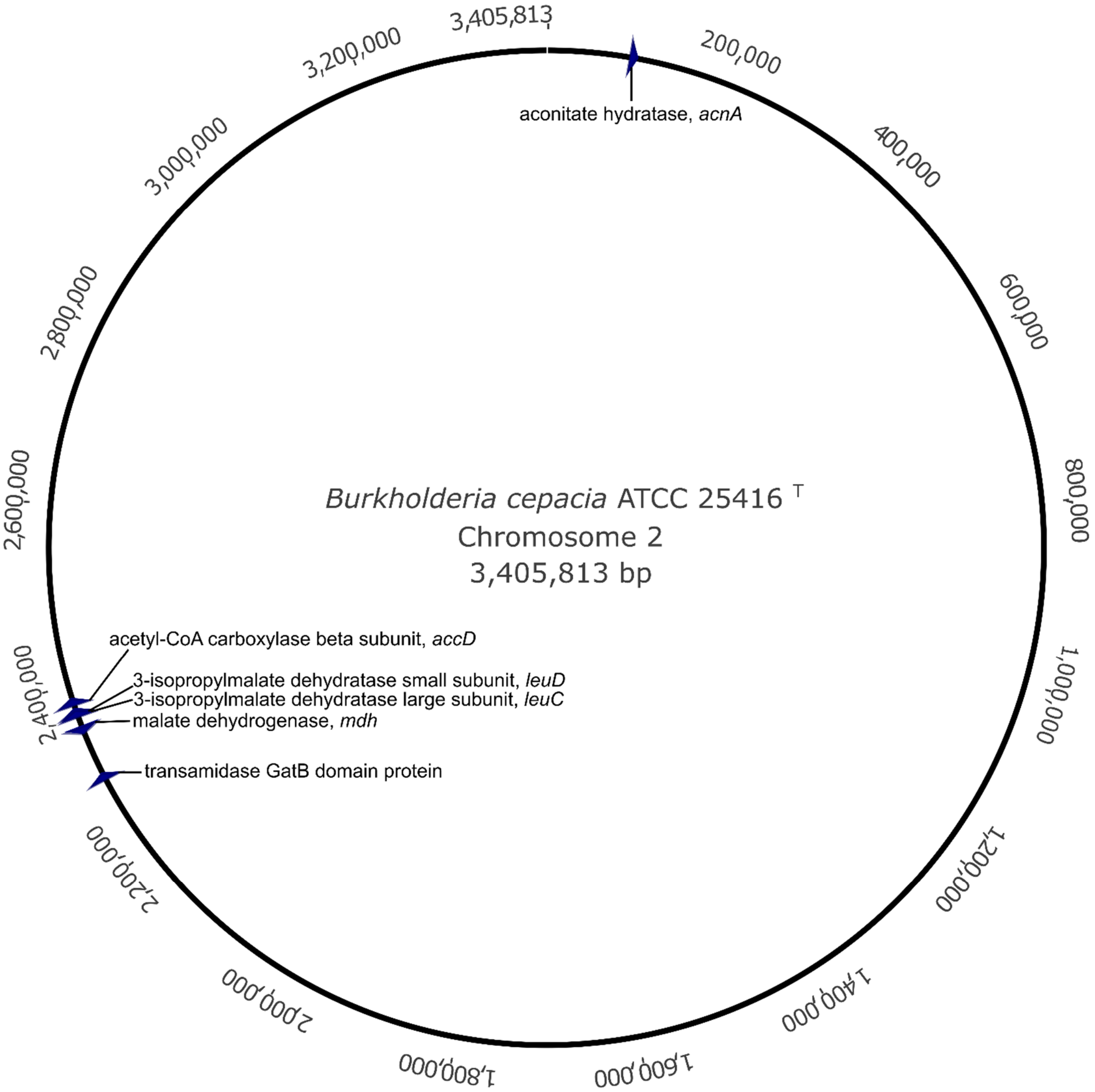

Supplementary Figure S2. The chromosomal positions of the 5 conserved genes present on chromosome 2, using *B. cepacia* ATCC 25416 ^{T} as reference. For specific positions, gene names and additional information, please refer to Supplementary Table S1.
