# Supplementary material for: Genome Data Provides High Support for Generic Boundaries in Burkholderia Sensu Lato
Source: Front Microbiol. 2017 Jun 26;8:1154. doi: 10.3389/fmicb.2017.01154 (PMC5483467; doi:10.3389/fmicb.2017.01154)
Supplement: Supplementary file 8 [file Image_3.PDF]

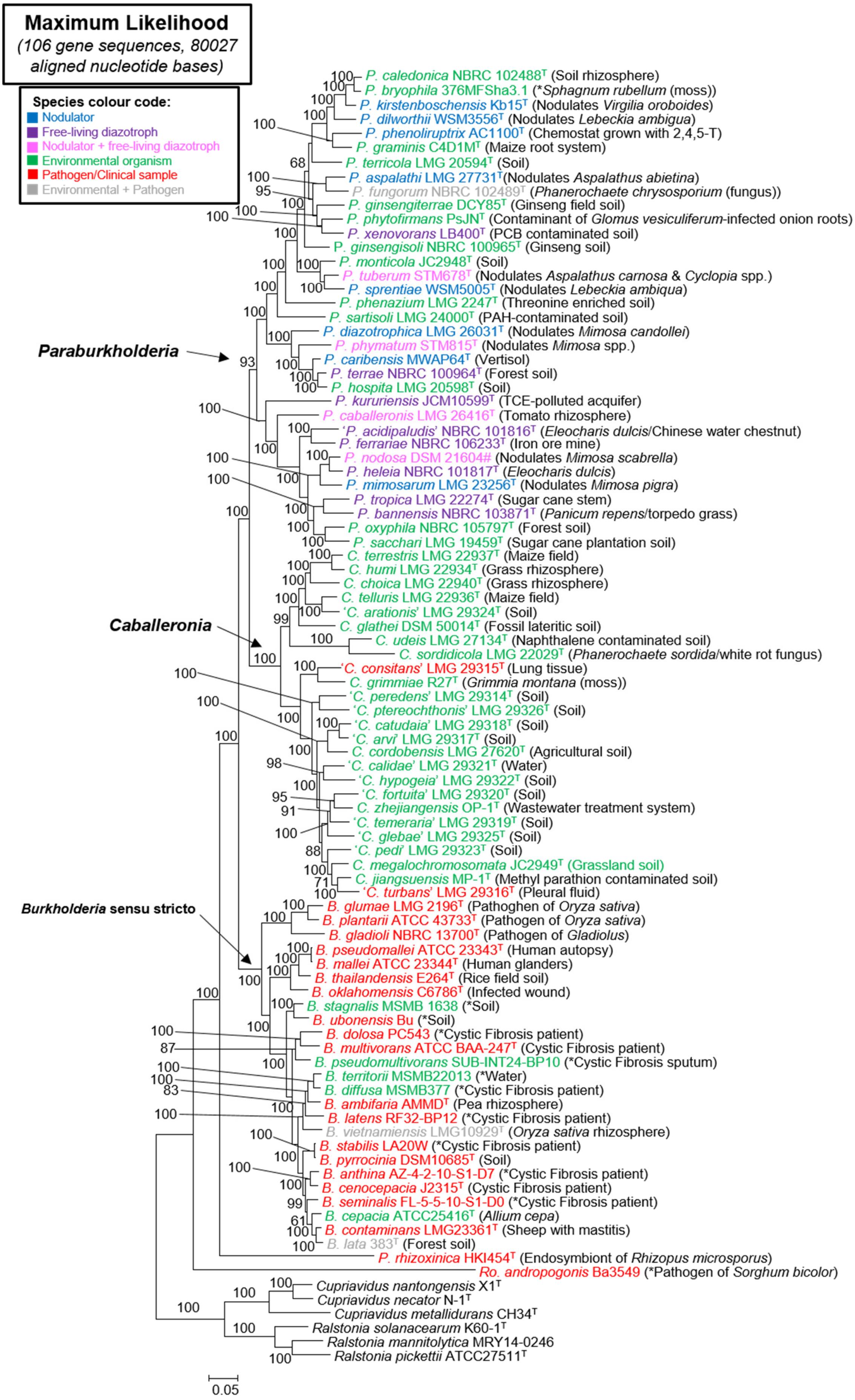

Maximum Likelihood
(106 gene sequences, 80027 aligned nucleotide bases)
Species colour code:
Nodulator
Free-living diazotroph
Nodulator + free-living diazotroph
Environmental organism
Pathogen/Clinical sample
Environmental + Pathogen
Paraburkholderia
Caballeronia
Burkholderia sensu stricto
P. caledonica NBRC 102488T (Soil rhizosphere)
P. bryophila 376MFSha3.1 (*Sphagnum rubellum (moss))
P. kirstenboschensis Kb15T (Nodulates Virgilia oroboides)
P. dilworthii WSM3556T (Nodulates Lebeckia ambigua)
P. phenoliruptrix AC1100T (Chemostat grown with 2,4,5-T)
P. graminis C4D1MT (Maize root system)
P. terricola LMG 20594T (Soil)
P. aspalathi LMG 27731T (Nodulates Aspalathus abietina)
P. fungorum NBRC 102489T (Phanerochaete chrysosporium (fungus))
P. ginsengiterrae DCY85T (Ginseng field soil)
P. phytofirmans PsJNT (Contaminant of Glomus vesiculiferum-infected onion roots)
P. xenovorans LB400T (PCB contaminated soil)
P. ginsengisoli NBRC 100965T (Ginseng soil)
P. monticola JC2948T (Soil)
P. tuberum STM678T (Nodulates Aspalathus carnosa & Cyclopia spp.)
P. sprentiae WSM5005T (Nodulates Lebeckia ambiqua)
P. phenazium LMG 2247T (Threonine enriched soil)
P. sartisoli LMG 24000T (PAH-contaminated soil)
P. diazotrophica LMG 26031T (Nodulates Mimosa candollei)
P. phymatum STM815T (Nodulates Mimosa spp.)
P. caribensis MWAP64T (Vertisol)
P. terrae NBRC 100964T (Forest soil)
P. hospita LMG 20598T (Soil)
P. kururiensis JCM10599T (TCE-polluted acquifer)
P. caballeronis LMG 26416T (Tomato rhizosphere)
'P. acidipaludis' NBRC 101816T (Eleocharis dulcis/Chinese water chestnut)
P. ferrariae NBRC 106233T (Iron ore mine)
P. nodosa DSM 21604# (Nodulates Mimosa scabrella)
P. heleia NBRC 101817T (Eleocharis dulcis)
P. mimosarum LMG 23256T (Nodulates Mimosa pigra)
P. tropica LMG 22274T (Sugar cane stem)
P. bannensis NBRC 103871T (Panicum repens/torpedo grass)
P. oxyphila NBRC 105797T (Forest soil)
P. sacchari LMG 19459T (Sugar cane plantation soil)
C. terrestris LMG 22937T (Maize field)
C. humi LMG 22934T (Grass rhizosphere)
C. choica LMG 22940T (Grass rhizosphere)
C. telluris LMG 22936T (Maize field)
'C. arationis' LMG 29324T (Soil)
C. glathei DSM 50014T (Fossil lateritic soil)
C. udeis LMG 27134T (Naphthalene contaminated soil)
C. sordidicola LMG 22029T (Phanerochaete sordida/white rot fungus)
'C. consitans' LMG 29315T (Lung tissue)
C. grimmiae R27T (Grimmia montana (moss))
'C. peredens' LMG 29314T (Soil)
'C. ptereochthonis' LMG 29326T (Soil)
'C. catudaia' LMG 29318T (Soil)
'C. arvi' LMG 29317T (Soil)
C. cordobensis LMG 27620T (Agricultural soil)
'C. calidae' LMG 29321T (Water)
'C. hypogeia' LMG 29322T (Soil)
'C. fortuita' LMG 29320T (Soil)
C. zhejiangensis OP-1T (Wastewater treatment system)
'C. temeraria' LMG 29319T (Soil)
'C. glebae' LMG 29325T (Soil)
'C. pedi' LMG 29323T (Soil)
C. megalochromosomata JC2949T (Grassland soil)
C. jiangsuensis MP-1T (Methyl parathion contaminated soil)
'C. turbans' LMG 29316T (Pleural fluid)
B. glumae LMG 2196T (Pathoghen of Oryza sativa)
B. plantarii ATCC 43733T (Pathogen of Oryza sativa)
B. gladioli NBRC 13700T (Pathogen of Gladiolus)
B. pseudomallei ATCC 23343T (Human autopsy)
B. mallei ATCC 23344T (Human glanders)
B. thailandensis E264T (Rice field soil)
B. oklahomensis C6786T (Infected wound)
B. stagnalis MSMB 1638 (*Soil)
B. ubonensis Bu (*Soil)
B. dolosa PC543 (*Cystic Fibrosis patient)
B. multivorans ATCC BAA-247T (Cystic Fibrosis patient)
B. pseudomultivorans SUB-INT24-BP10 (*Cystic Fibrosis sputum)
B. territorii MSMB22013 (*Water)
B. diffusa MSMB377 (*Cystic Fibrosis patient)
B. ambifaria AMMDT (Pea rhizosphere)
B. latens RF32-BP12 (*Cystic Fibrosis patient)
B. vietnamiensis LMG10929T (Oryza sativa rhizosphere)
B. stabilis LA20W (*Cystic Fibrosis patient)
B. pyrrocinia DSM10685T (Soil)
B. anthina AZ-4-2-10-S1-D7 (*Cystic Fibrosis patient)
B. cenocepacia J2315T (Cystic Fibrosis patient)
B. seminalis FL-5-5-10-S1-D0 (*Cystic Fibrosis patient)
B. cepacia ATCC25416T (Allium cepa)
B. contaminans LMG23361T (Sheep with mastitis)
B. lata 383T (Forest soil)
P. rhizoxinica HKI454T (Endosymbiont of Rhizopus microsporus)
Ro. andropogonis Ba3549 (*Pathogen of Sorghum bicolor)
Cupriavidus nantongensis X1T
Cupriavidus necator N-1T
Cupriavidus metallidurans CH34T
Ralstonia solanacearum K60-1T
Ralstonia mannitolytica MRY14-0246
Ralstonia pickettii ATCC27511T
0.05

Supplementary Figure S3. Maximum-likelihood phylogeny of the nucleotide sequences of 106 conserved genes for the 92 strains investigated in this study. The partitioned dataset employed independent model parameter estimates for the General Time Reversible model (GTR; Tavaré, 1986) for each partition and was analysed in RAxML v 8.2 (Stamatakis, 2014). Values indicated at nodes were inferred for 1000 bootstrap pseudo-replicate datasets.
